# Prognostic value of *OCT4A* and *SPP1C* transcript variant co-expression in early-stage lung adenocarcinoma

**DOI:** 10.1186/s12885-020-06969-0

**Published:** 2020-06-05

**Authors:** Seijiro Koshimune, Mitsuko Kosaka, Nobuhiko Mizuno, Hiromasa Yamamoto, Tomoyuki Miyamoto, Kohta Ebisui, Shinichi Toyooka, Aiji Ohtsuka

**Affiliations:** 1 Department of Human Morphology, Okayama University Graduate School of Medicine, Dentistry and Pharmaceutical Sciences, 2-5-1, Shikata-cho, Kita-ku, Okayama, 700-8558 Japan; 2 Department of General Thoracic Surgery and Breast and Endocrinological Surgery, Okayama University Graduate School of Medicine, Dentistry and Pharmaceutical Sciences, Okayama, Japan; 3 Department of Medical Life Sciences, Cancer Cell Research Institute, Kyushu University of Health and Welfare, Yoshino-cho, Nobeoka, Miyazaki, Japan

**Keywords:** OCT4, SPP1, lung adenocarcinoma, tumour-initiating cell, cancer stem cell, cell migration

## Abstract

**Background:**

Octamer-binding transcription factor 4A (OCT4A) is essential for cell pluripotency and reprogramming both in humans and mice. To date, however, the function of human OCT4 in somatic and/or tumour tissues is largely unknown.

**Methods:**

RT-PCR was used to identify full-length splice forms of *OCT4* transcripts in normal and cancer cells. A FLAG-tagged OCT4 genomic transgene was used to identify OCT4-positive cancer cells. A potential role for OCT4 in somatic cancer cells was examined by cell ablation of OCT4-positive cells using promoter-driven diphtheria toxin A. *OCT4* and secreted phosphoprotein 1 (*SPP1*) transcripts in early-stage lung adenocarcinoma tumours were analysed and compared with pathohistological features.

**Results:**

The results show that, unlike in murine cells, *OCT4A* and *OCT4B* variants are transcribed in both human cancer cells and in adult tissues such as lung, kidney, uterus, breast, and eye. We found that *OCT4A* and *SPP1C* are co-expressed in highly aggressive human breast, endometrial, and lung adenocarcinoma cell lines, but not in mesothelial tumour cell lines. Ablation of OCT4-positive cells in lung adenocarcinoma cells significantly decreased cell migration and *SPP1C* mRNA levels. The OCT4A/SPP1C axis was found in primary, early-stage, lung adenocarcinoma tumours.

**Conclusions:**

Co-expression of OCT4 and SPP1 may correlate with cancer aggressiveness, and the OCT4A/SPP1C axis may help identify early-stage high-risk patients with lung adenocarcinoma. Contrary to the case in mice, our data strongly suggest a critical role for OCT4A and SPP1C in the development and progression of human epithelial cancers.

## Background

Cancer stem cells (CSCs) or tumour-initiating cells (TICs) [1, 2] are essential for understanding drug resistance, tumour relapse, metastasis, and tumour cell behaviour in clinical treatments. Single-cell mRNA sequencing is a powerful technology for understanding cellular heterogeneity in normal and tumour tissues and has enhanced our understanding of rare cells that might affect drug resistance and relapse in cancer treatment; however, this method has not yet been optimised for the detection of genes expressed at low levels, such as transcription factors [3, 4].

The *POU class 5 homeobox 1* (*POU5F1*)*/octamer-binding transcription factor 4A* (*OCT4*) gene encoding the octamer-binding transcription factor (also known as OCT3A and OCT3/4A) is particularly crucial for cell pluripotency in early embryonic development and propagation of the mammalian germline [5–9]. Further, it is a marker for germ-cell tumours and a potential CSCs marker [6, 10]. *OCT4* is known to be involved in the translocation with the Ewing’s sarcoma gene on chromosome 21, leading to tumorigenesis in humans [11, 12]. Another study reported the identification of CSC-like phenotype by OCT4 promoter mediated activity in an osteosarcoma cell line [13]. Although these studies suggest that OCT4 plays an role in human somatic cancers, its somatic function is controversial. Since its proposed role is based on the results derived from multiple transcript variants and related, active pseudogenes, this may have introduced false positives and led to an erroneous or questionable interpretation of the data [14–16]. In addition, previous studies also indicated that OCT4A does not play a functional role in adult somatic murine tissues [17, 18], therefore, many researchers have been reticent to accept a role for OCT4A in human adult somatic tissues or related cancers [14, 18–22].

In our previous study, we developed a highly specific reverse transcription polymerase chain reaction (RT-PCR) assay to analyse the human *OCT4* gene, which eliminated false positives and identified multiple transcripts in human carcinoma cell lines [16]. Additionally, we reported that OCT4 was translated in a subpopulation of human endometrial cancer cells characterised by enhanced cell migration and invasion [16]. Consistent with our findings, another group reported that endogenous OCT4A functions as a transcription factor in somatic cancer cells [23]. These results renew the discussion surrounding a critical role for OCT4A or other OCT4 variants in human somatic cancers and germ-cell tumours. To our knowledge, variant-specific expression of *OCT4* transcripts could not be assessed using currently available high-throughput databases [18, 24]; therefore, in the present study, we explored the potential of multiple *OCT4* transcript variants to act as prognostic biomarkers in human somatic cancers.

Secreted phosphoprotein 1 (SPP1) [also designated as osteopontin (OPN) [25, 26]] mediates critical processes involved in cancer progression, including immune response, cell adhesion and migration, and tumourigenesis [27–29]. Three major *SPP1*-transcript splice variants exist that encode SPP1A (the full-length isoform), SPP1B (lacking exon 5), and SPP1C (lacking exon 4). Each SPP1 isoform is characterised as a potential prognostic marker for multiple malignancies, such as lung, breast, and ovarian cancers [28]; however, they cannot be used as universal markers for cancer prognosis based on the variance in their expression and associated signalling among tissues [30]. We hypothesised that various *OCT4*- and *SPP1*-transcript variants might be associated with the development and aggressiveness of various human somatic cancers; therefore, we comprehensively assessed the expression of these variants in healthy and cancerous human tissues in order to characterise their potential biological and clinical roles in human lung adenocarcinoma (LUAD), which is the leading cause of cancer-related deaths worldwide [31].

## Methods

### Cell culture

Lung cancer cell lines (HCC827, HCC827GRH2, HCC827ARH, H1299, PC-9, HCC4006, and H1975), mesothelioma cell lines (MSTO-211H and H2052), and immortalised cell lines (HBEC-5KT and MeT-5A) were provided and described by the co-authors [32]. Other cell lines were obtained from Japanese Collection of Research Bioresources (JCRB, Osaka, Japan), American Type Culture Collection (ATCC, Manassas, VA) and European Collection of Authenticated Cell Cultures (ECACC, Salisbury, UK). MCF7 (JCRB0134), HEC50B (JCRB1145), MDAMB231 (ATCC HTB-26™), PA-1 (ATCC CRL-1572™) and Ishikawa (ECACC99040201) were purchased in 2017 or later and used in this study. All cell types are summarized in supplementary information **(**Table S1). RNA extraction from these cell lines was performed within 2 weeks from receipt and not exceeding 3 passages. Cells for bioassay were for mycoplasma contamination and passaged for < 6 months prior to experimentation. For monolayer culture, cells were maintained in culture dishes or plates (Sumitomo Bakelite Co. Ltd., Shinagawa, Japan) in Dulbecco’s modified Eagle medium (DMEM) supplemented with 8% foetal bovine serum. Sphere-formation assays were performed as previously described [32]. Briefly, a total of 5 × 10 [3] single, dissociated cells were prepared per 24-well plate with an ultra-low attachment surface (Corning, Corning, NY, USA) and incubated for 14 days in DMEM/F12 medium supplemented with B29 supplement (Thermo Fisher Scientific, Waltham, MA, USA), epidermal growth factor (20 ng/mL; PeproTech, Rocky Hill, NJ, USA), and fibroblast growth factor 2 (10 ng/mL; PeproTech). The number of spheres > 150 μm in diameter was microscopically counted per well.

### Experimental animals and ethics statement

C57BL/6 N mice (4-months and 3-days old) were obtained from Japan SLC Co. (Shizuoka, Japan) and housed in rooms maintained at constant temperature and humidity with a 12 h light/dark cycle. For this study, tissues were obtained from mice that had to be killed owing to excessive breeding. This study was approved by the Animal Care and Use Committee of RIKEN and Okayama University and performed in accordance with the Japanese Council on Animal Care guidelines (No. AH15–09-5, No. OKU-2012259). All animal procedures were performed in accordance with approved protocols.

### Total RNA extraction and cDNA preparation

Total RNA samples from normal human tissues (Table S2) were purchased from Agilent Technologies (BioChain; Santa Clara, CA, USA), Clontech (Takara, Shiga, Japan), and Zyagen (San Diego, CA, USA). Clinical tumour tissues were stored in RNAlater stabilisation solution (Thermo Fisher Scientific). We used the total RNA samples from 3-days old mice, which we had analysed before [18]. Total RNA from fresh tissues was extracted using ISOGENE II (Nippon Gene Co. Ltd., Toyama, Japan) and immediately homogenised (Beads Crusher μT-12; TAITEC, Saitama, Japan) and processed according to the manufacturer’s instructions.

### RT-PCR

cDNA was synthesised from 0.5 μg RNA using oligo-dT primers and a PrimeScript II first-strand cDNA synthesis kit (#6210A/B; Takara) according to the manufacturer’s instructions. Reverse transcriptase negative controls were used to assess genomic DNA contamination. The primers used for PCR are described in Table S3 [16]. We used two PCR enzymes [EmeraldAmp PCR Master Mix, RR300A; Takara) or PrimeSTAR HS DNA polymerase (R010A; Takara)] in a total volume of 20 μL. Thermal cycling conditions for *OCT4* were; 35 cycles at 96 °C for 30 s and 68 °C for 2 min. Those for *SPP1* were 30 cycles at 96 °C for 30 s and 68 °C for 1 min. The PCR products (~ 20%) were separated on a 1.5% agarose gel, stained with ethidium bromide, and visualised under ultraviolet light (312-nm wavelength). Images were captured using a GelPrint 2000i system (Genomic Solutions, Ann Arbor, MI, USA).

### Cloning and sequencing analysis

After electrophoretic separation, PCR amplicons were extracted using the QIAquick gel extraction kit (#28706; Qiagen, Hilden, Germany) and cloned into the pCR-Blunt vector (#K280040; Life Technologies, Carlsbad, CA, USA). Isolated plasmids were analysed on an ABI3130 sequencer (Central Research Laboratory, Okayama University Medical School, Okayama, Japan) using M13 forward and reverse primers. Plasmids extracted from randomly selected colonies were classified based on their sequences, which were analysed using BLAST (https://blast.ncbi.nlm.nih.gov/Blast.cgi) or a genetic information processing software (Genetyx Corporation, Tokyo, Japan).

### Wound-healing assay

Wound-healing migration assays were performed according to the manufacturer’s instructions (#80209; Ibidi GmbH, Martinsried, Planegg, Germany). Transfected cells (2.5 × 10^4^) were seeded into each well, and the culture insert was gently removed after 24 h. Images were captured over time post-wounding, and the wound area was calculated using ImageJ software (NIH, Bethesda, MD, USA).

### Plasmid construction and transfection

Plasmids were constructed as previously described [16]. Briefly, a *POU5F1* genomic DNA fragment (− 5000 to + 10,784 bp) was subcloned from BAC clone RPCI-11-1058 J10 using a BAC subcloning kit (Gene Bridges, Heidelberg, Germany) to create pOCT4Gen. An in-frame FLAG tag was added to the 3′ end of the exon 5 open reading frame to generate pOCT4Gen-FLAG. The backbone of this vector contained a CMV promoter-driven red fluorescent protein (RFP) for visualisation of the transfected cells. A *Nco*I fragment of pOCT4Gen (− 4733 to + 77 bp) was ligated into the *Nco*I site of the diphtheria toxin fragment A (DT-A) vector and an enhanced GFP (EGFP) vector to construct pOCT4-DTA and pOCT4-GFP, respectively. Plasmid DNA transfection was performed using Lipofectamine 2000 or 3000 (Life Technologies, Carlsbad, CA, USA) according to the manufacturer’s instructions. Cells were transfected with pOCT4-GFP or pOCT4-DTA and incubated for 24 h to assess the biological effects of OCT4-positive cell ablation. The same number of viable cells was used for each assay. The study was conducted in accordance with Okayama University Safety Committee for Recombinant DNA Experiments guidelines.

### Immunocytochemistry

Non-transfected or transfected cells were fixed in 4% paraformaldehyde for 15 min at 25 °C and permeabilised in 0·2% Triton X-100 for 20 min. Cells were incubated with mouse monoclonal anti-FLAG antibodies (#F1804; Sigma-Aldrich, St. Louis, MO, USA) at 25 °C for 45 min, washed thrice in phosphate-buffered saline (PBS), incubated with secondary antibodies (Alexa 488-conjugated goat anti-mouse IgG; #ab150113; Invitrogen, Carlsbad, CA, USA), and washed thrice in PBS. The cells were then counterstained with 4′,6-diamidino-2-phenylindole (#D1306; Thermo Fisher Scientific) and visualised under a fluorescence microscope (Zeiss Axiovert135, Germany). PBS was used instead of the primary antibody in negative controls.

### Transwell migration assay

Cell migration assays were performed using 24-well cell culture inserts with 8-μm pores (#354480; Corning). Transfected cells (2.5 × 10^4^) were seeded into the top chambers of the inserts, and normal growth medium was added to the lower chambers. After 24 h, cells remaining in the upper chamber were removed, and those attached to the underside of the membrane in the lower chamber were fixed with methanol, stained with 10% Giemsa solution (#15003; Muto Pure Chemicals, Tokyo, Japan), and counted under a light microscope. Five representative fields per well and three replicate wells per condition were analysed.

### Real-time quantitative PCR (qPCR) for *SPP1C*

cDNA was synthesised from 0.5 μg RNA using random decamer primers for qPCR analysis. Gene expression analysis was carried out utilizing KAPA SYBR FAST Universal 2× qPCR Master Mix (Kapa Biosystems, Boston, USA) on a Step One Plus real time PCR system (Thermo Fisher Scientific) according to the manufacturer’s protocol. PCR amplification conditions were: 95 °C for 2 min, followed by 40 cycles of 95 °C for 5 s and 60 °C for 30 s, and melting-curve analysis (60–95 °C with a heating rate of 0.3 °C/s and continuous fluorescence measurement). Relative gene expression of *SPP1C* was evaluated using the ΔΔCt method and normalisation to 18S ribosomal RNA or beta-actin. Sequences of these PCR primer sets were described in Table S3.

### Analysis of clinical tissue samples

All LUAD patients were stage I at the time of diagnosis. Tumour tissues were collected during surgical resections (2010–2016) and snap-frozen at − 80 °C or stored in RNAlater solution (Qiagen) until RNA extraction. LUAD patient samples (77 cases) were collected from Okayama University Hospital, and high-quality RNA was extracted from 58 tumour specimens. Clinicopathological data for each patient were obtained retrospectively from the medical records of Okayama University Hospital Biobank (Okadai Biobank; Ref. No. OC17003), and histopathologic review of tumour specimens was conducted at Okayama University Hospital. Clinical and histopathological characteristics are described in Table S5. This study (No. K1612–023) was approved by the ethical committee of the Okayama University Graduate School of Medicine, Dentistry, and Pharmaceutical Sciences, (Okayama, Japan).

### Statistics

Data represent the mean ± standard deviation (SD) of at least three independent experiments. Statistical evaluation was conducted by paired, two-tailed Student’s *t*-test and correlations between *OCT4A* and *SPP1C* expression in LUAD tumours were analysed using Fisher’s exact test. Differences with *P* < 0.05 were considered statistically significant.

## Results

### *OCT4*-transcript variants are expressed in human somatic tissues at lower levels than in *PA-1,* a human ovarian teratocarcinoma cell line

**Fig. 1:**
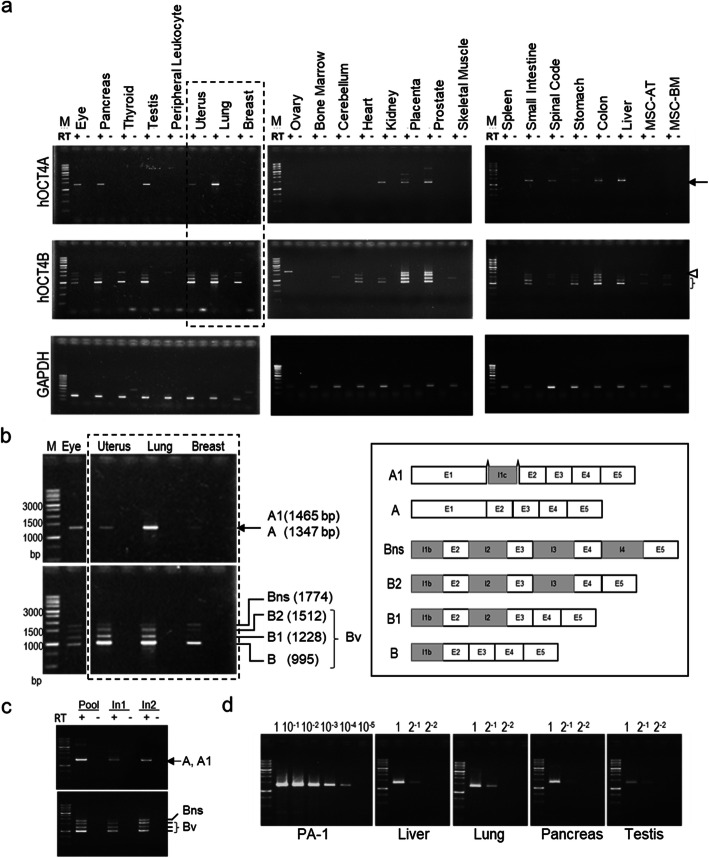
*OCT4A-* and *OCT4B*-transcript variants are expressed in various human somatic tissues. **a** Gel electrophoresis of PCR products derived from primer sets specific for human *OCT4A-* and *OCT4B*-transcript variants and *GAPDH* (loading control). RT+ and RT− indicate treatments with and without treatment of reverse transcriptase, respectively. Detailed information on RNA sources is presented in Table S2. Three independent experiments were performed, and representative images are shown. Arrow, *OCT4A*; arrowhead: *OCT4Bns*; bracket: *OCT4B*v (B-splice variants other than OCT4Bns). **b** The size and position of expected bands are indicated in a partially expanded image surrounded by a dashed line (left). Schematic diagram of human *OCT4* mRNA variant structures (right). **c** Expression of human *OCT4A-* and *OCT4B*-transcript variants in the pooled sample and in two samples of normal adult lung tissues from different sources. **d** Semi-quantitative comparison of human *OCT4A-*expression levels between PA-1 cells and adult tissues (liver, lung, pancreas, and testis). A 10-fold series of dilutions of cDNA from PA-1 cells were used for standard PCR, and a 2-fold series of dilutions of cDNA from human tissue samples were used for comparison of PCR samples. All full-length gels are presented in Supplementary Figs. S4–1-4

We examined the expression of *OCT4*-transcript variants in 24 commercially available RNA samples from human tissues and cells using highly specific primer sets [16] (Table S3). Consistent with our previous findings [16], the PCR and sequencing analysis revealed that the primer sets specifically amplified the desired target cDNA; however, faint bands for *glyceraldehyde 3-phosphate dehydrogenase* (*GAPDH*) in thyroid and breast samples indicated minor genomic DNA contamination (Fig. 1a).

Unexpectedly, *OCT4A* and *OCT4B* variant transcripts were readily detected in testis, ovary, and 13 other somatic tissues (eye, pancreas, uterus, lung, breast, kidney, placenta, prostate, small intestine, spinal cord, stomach, colon, and liver) (Fig. 1a). A schematic diagram of human *OCT4A-* and *OCT4B*-transcript variant structures and the size of the expected PCR products are displayed in Fig. 1b [16]. Sequencing analysis of the PCR products revealed that they were derived from human *OCT4A* and *OCT4A1*, as well as from at least four different human *OCT4B*-transcript variants (*OCT4B, OCT4B1, OCT4B2,* and *OCT4Bns*) (Table S4). *OCT4A1*, a minor transcript variant, has a 118-bp insertion in exon 1C and located between 3569- and 3687-bp downstream of the exon 1/intron 1 boundary, which encodes an in-frame endogenous TGA stop codon, as described previously [16]. Therefore, we focused only on *OCT4A*, the full-length form of the variants, in this study.

Only *OCT4B* variants were readily detectable in the thyroid, peripheral leukocytes, cerebellum, heart, skeletal muscle, and two kinds of mesenchymal stem cells derived from the adipose tissue (MSC-AT) or bone marrow tissue (MSC-BM) (Fig. 1a). In peripheral leukocytes, bone marrow, and spleen, neither *OCT4A* nor *OCT4B* variants were detectable (Fig. 1a), suggesting that *OCT4*-transcript variants are differentially expressed in adult human somatic tissues.

Next, we sought to examine whether *OCT4*-transcript variants were ubiquitously or selectively expressed in certain individuals. Using commercially available total RNA isolated from the lungs of two separate donors, we found that the same *OCT4A-* and *OCT4B*-transcript variants were expressed in the lungs of both individuals (Fig. 1c). The RNA samples isolated from the eye, thyroid, pancreas, kidney, skeletal muscle, and stomach (Fig. 1a) were also from a single donor, and all of these samples expressed *OCT4B*, whereas except for the skeletal muscle sample, all others expressed *OCT4A*. Collectively, these results indicated that *OCT4* expression does not exhibit person-to-person variation and is ubiquitously expressed in certain human somatic tissues.

Notably, *OCT4A*-expression levels in the liver, lung, and pancreas were similar to or higher than those in the testis (Fig. 1a). Therefore, we further examined *OCT4A*-expression levels in these tissues by semi-quantitative RT-PCR. Comparison of the band intensities of the PCR products with serial dilutions of cDNA derived from RNA isolated from PA-1 cells (Fig. 1d) revealed that band intensities associated with *OCT4A* in the adult tissues were 1/1000- to 1/10000-times lower than *OCT4A* in PA-1 cells. This might indicate that *OCT4A* transcripts are highly expressed by a small subpopulation of cells in certain adult somatic tissues, where their expression might be functionally significant.

### *Oct4*-transcript variants are not expressed in adult murine somatic tissues

To date, no functional roles for OCT4A in murine, somatic, normal, or tumour tissues have been substantiated [17, 18]. In a previous report, we found no *Oct4A*-transcript variants but instead identified novel transcript variants (*Oct4B* and *Oct4C*) in postnatal murine tissues [18]. Based on the *OCT4* expression observed in human somatic tissues (Fig. 1), we re-examined the expression of *Oct4*-transcript variants in 4-month-old (adult) mouse tissues using a previously [18] and newly developed PCR primer set (Table S3). It was confirmed that *Oct4A* and *Oct4B* variants are expressed in testis but not in adult murine somatic tissues (Fig. S1). This clear difference in the expression of *OCT4*/*Oct4* transcripts in human and murine somatic tissues reveals the potential inadequacy of using a mouse model to characterise the somatic function of human OCT4.

### Expression of *OCT4*-transcript variants in malignant and non-malignant cells of human somatic tissues

**Fig. 2:**
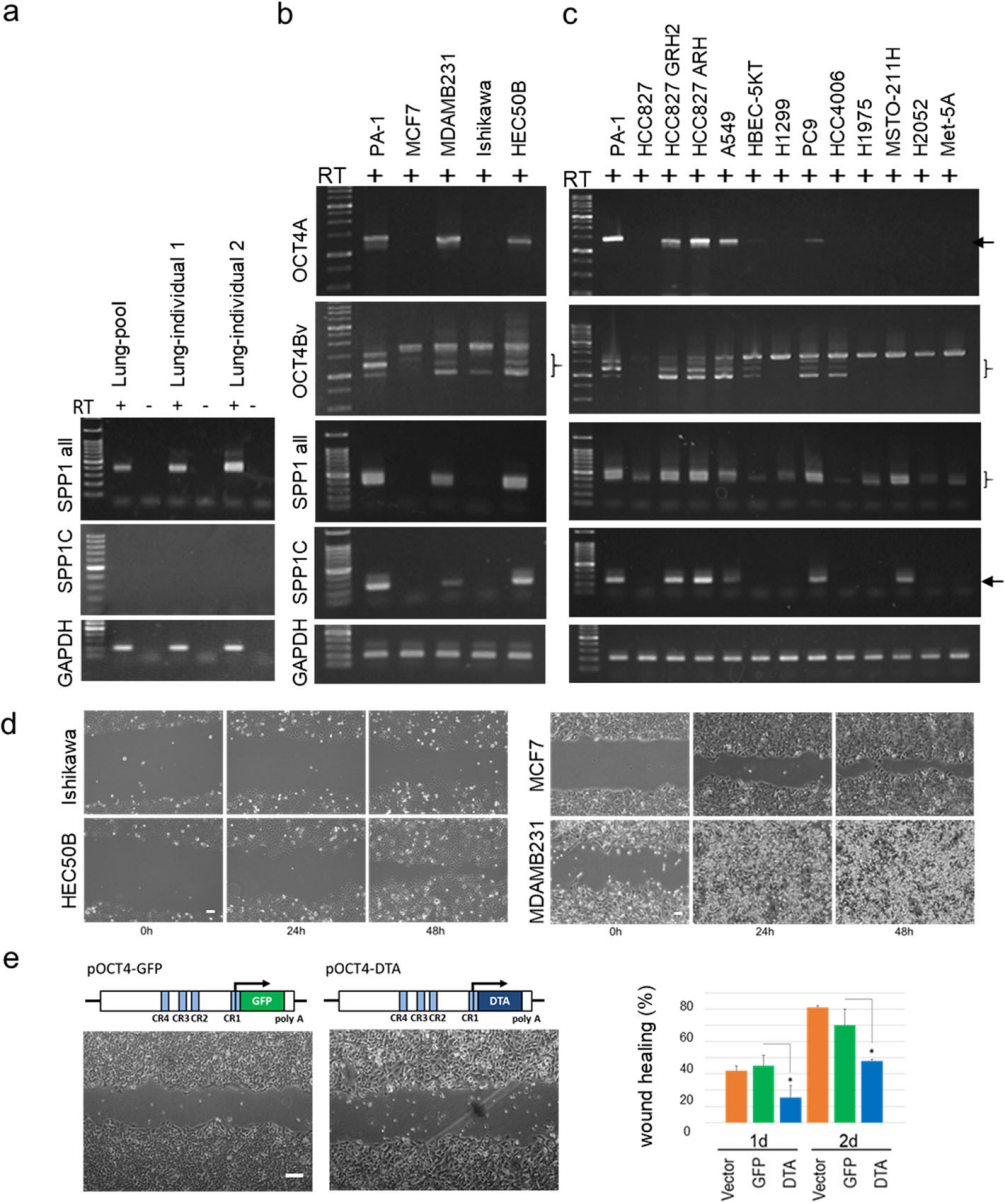
*OCT4* and *SPP1* expression in human various cell lines and OCT4 in cell migration. Gel electrophoresis of PCR products using primer sets specific to human *OCT4A*, *OCT4Bv*, *SPP1-all*, *SPP1C,* and *GAPDH* (positive controls). RT-PCR using specific primer sets for detecting *OCT4A* and *OCT4B* variants and *SPP1* variants, respectively (Table S3). RT+ and RT− indicate treatments with and without reverse transcriptase, respectively. Arrowheads, *OCT4Bns* bands; and asterisks, *OCT4B*-splice variants (*OCT4B, OCT4B1*, and *OCT4B2*; denoted as *OCT4Bv*). **a** Pooled RNA samples from two patients and human lung tissues (Table S2). **b**–**d** Representative bright-field images of wound-healing experiments using endometrial and breast cancer cell lines (Ishikawa vs HEC50B; and MCF7 vs MDA-MD-231) acquired at 24- and 48-h post-wounding. Scale bar: 100 μm. **e** Representative bright-field images of A549 cells transfected with pOCT4-DTA or pOCT4-GFP control plasmids and acquired at 12-h post-wounding. The migration rate is expressed as the percentage of wound-closure area. Data represent the mean ± standard deviation of the closure area at each time point from three independent experiments. *P < 0.05compared with the control. Scale bar: 100 μm. All full-length gels are presented in Supplementary Figs. S5–1-5

In a prior analysis [16], we found that only *OCT4Bns* (a no-splice form of the *OCT4B* transcript) was expressed in normal human tissue-derived cell lines and certain cancer cells. The function of OCT4Bns remains unknown, but its expression does not appear to correlate with the expression of other splice variants (*OCT4A* and/or *OCT4Bv*). Moreover, we reported that multiple *OCT4A* and *OCT4Bv* transcripts were readily detected in A549, an aggressive human lung cancer line, and in HEC50B, an aggressive endometrial cancer cell line; however, only a few transcript variants were detected in Ishikawa cells, a non-aggressive endometrial cancer cell line [16]. In the present study, to confirm a potential role for OCT4A and OCT4Bv (B-splice variants save for OCT4Bns) in the aggressiveness of human somatic cancers, we examined the expression of *OCT4* transcripts in breast and various lung tumour cell lines (Fig. 2b and c).

We observed that the predicted *OCT4A* and *OCT4Bv* PCR products were readily detected in MDA-MB-231, a highly aggressive breast cancer cell line (Fig. 2b). Conversely, consistent with our previous data, *OCT4A* and *OCT4Bv* transcripts were undetectable or expressed at very low levels in MCF7, a non-aggressive cancer cell line (Fig. 2b) [16], suggesting that the expression of these *OCT4* transcripts is positively correlated with the aggressiveness of breast and endometrial cancer cells, which is inconsistent with a previous bioinformatics analysis [24].

Similarly, in highly aggressive lung cancer cell lines (HCC827 GRH2, HCC827 ARH, and PC9), as well as A549, we clearly detected *OCT4A* and *OCT4Bv* transcripts (Fig. 2c), whereas their transcripts were undetectable in less aggressive cancer cell lines (HCC827 and H1975). Moreover, in a non-aggressive lung cancer line (HCC4006) and an immortalised bronchial epithelial cell line (HEBC-5KT), we detected *OCT4Bv* but not *OCT4A* transcripts. Notably, *OCT4A* and *OCT4Bv* transcripts were not detected in mesothelial tumour cell lines (MSTO-211H and H2052), a non-malignant mesothelial cell line (Met-5A), or an aggressive large-cell lung cancer line (H1299) that is p53-null [31]. The absence of *OCT4*-transcript expression (except for the *OCT4Bns* variant) in these cell lines might result from lung cancer cells being of different origins. Alternatively, all analysed cell lines highly expressing *OCT4A* and *OCT4Bv* transcripts represent cancers of epithelial origin (Table S1). Based on these data, we hypothesised that *OCT4* splicing variants might be involved in the progression of certain epithelial cancers, including lung, uterus, and breast cancers.

### A possible role for OCT4 in cancer cell migration

Collective migration is a hallmark of cancer cell invasion and metastasis [33, 34]. Wound-healing assays revealed that MDA-MD-231 and HEC50B cells migrated faster than MCF7 and Ishikawa cells, respectively (Fig. 2d) [35]. We herein focused on the effect of OCT4 on the migration of aggressive A549 LUAD cells, because we previously reported a small subpopulation of OCT4-positive cells in A549 cells [16]. To assess the potential effect of OCT4 on collective migration, we evaluated the effects of DTA-mediated ablation of A549 cells. We transfected cells with plasmids using the *OCT4* promoter to express either DTA (pOCT4-DTA) or GFP (pOCT4-GFP) [16] and confirmed that cellular morphology and cell numbers were not significantly affected by transfection with either plasmid (Fig. 2e, left). We observed a significant difference in the migration of A549 cells transfected with pOCT4-DTA compared with those transfected with the control plasmid (pOCT4-GFP) (Fig. 2e). These data suggested a significant contribution of OCT4 to the migration and aggressiveness of A549 LUAD cells.

### OCT4- and SPP1-transcript variant expression is correlated in human cancer cell lines

SPP1 is a multifunctional cytokine that affects cell proliferation, survival, drug resistance, invasion, and stem-like behaviour and promotes the invasion and metastatic progression of many carcinomas [27–29]. Based on these effects, we proffered that there may be a possible association between the expression of *OCT4* and *SPP1* variants in cancer cells. We found significant expression of *SPP1, OCT4A,* and *OCT4Bv* transcripts in MDA-MB-231, HEC50B, and A549 (cancer cell lines) and in HCC827 GRH2 and HCC827 ARH (drug-resistant lung cancer cell lines) (Fig. 2b and c). Interestingly, we observed a significant correlation between *OCT4A* and *SPP1C* expression in the aggressive cancer cell lines (Fig. 2b and c). Notably, *OCT4A* and *SPP1C* were expressed at much lower levels in the parental cell line (HCC827) compared with the two drug-resistant cell lines (HCC827 GRH2 and HCC827 ARH) (Fig. 2c). Furthermore, very few (if any) *OCT4A* and *SPP1C* transcripts were detected in non-aggressive cancer lines (MCF7 and Ishikawa) (Fig. 2b). Like previous studies on the breast and ovary [35–37], *SPP1C* was not detected in adult normal lung tissues, although other *SPP1* variants were expressed (Fig. 2a). However, we confirmed the expression of *SPP1C* in several kinds of human adult non-tumour tissues (Fig. S2), supporting the idea that *SPP1C* alone cannot serve as a selective marker for cancer diagnosis [31]. Our results raise a new possibility that the OCT4A/SPP1C axis might closely relate to aggressiveness in certain human epithelial cancers.

### Characterisation of OCT4-positive cells in HCC827 and HCC827 GRH2 LUAD cell lines

HCC827 GRH2 is a gefitinib-resistant cell line derived from HCC827 LUAD cells [32] and it exhibits stem cell-like properties [32]. Previous cDNA microarray analysis showed that *ALDH1A* and *SPP1* expression accounts for the highest increase in HCC827 GRH2 cells relative to the parental cell line [32]. Alternatively, *OCT4* expression was not detected in the microarray data, suggesting that this analysis may be unsuitable for the detection of specific *OCT4A-*transcript variants in cancer cells [16]. Our highly specific method identified *OCT4A, OCT4Bv*, and *SPP1* transcripts in HCC827 GRH2 cells but not in parental HCC827 cells (Fig. 2c).

**Fig. 3:**
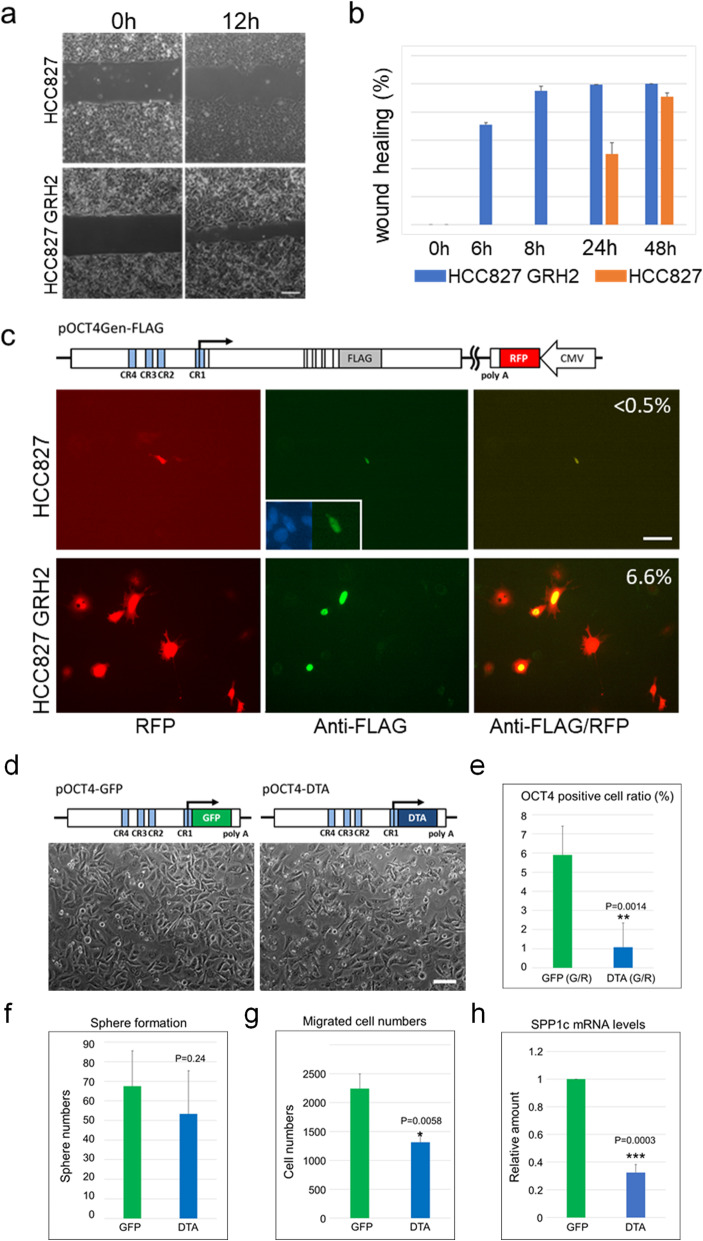
Characterisation of the OCT4-positive cell population in HCC827 and HCC827 GRH2 cells. **a** Representative bright-field images of wound-healing experiments acquired at 0- and 12-h post-wounding. Scale bar: 100 μm. **b** Migration rate was expressed as the percentage of wound-closure area. **c** Detection of possible OCT4 translation in HCC827 and HCC827 GRH2 cells using the FLAG-tagged genomic transgene (pOCT4Gen-FLAG) according to immunocytochemistry. RED: transfected RFP-positive cells; GREEN: FLAG-positive cells. The ratio of FLAG−/RFP-positive cells for each is indicated. Scale bar: 100 μm. **d–h** Effects of ablating OCT4-positive cells from HCC827 GRH2 cells. **d** Phase-contrast image of pOCT4-EGFP- or pOCT4-DTA-transfected HCC827 GRH2 cells at 24-h post-transfection. These cells were used for the assays described in **e–h**. **e** OCT4-positive cell rate among pOCT4-DTA-transfected HCC827 GRH2 cells. **f** Sphere-formation activities, **g** cell migration according to Transwell migration assays, and **h***SPP1C* mRNA levels by qPCR. Data represent the mean ± standard deviation from three independent experiments. *P < 0.05, **P < 0.005, ***P < 0.0005, compared with the control. Scale bar: 100 μm

We herein examined collective migration activity in HCC827 GRH2 cells compared with the parental HCC827 cells. The migration of HCC827 GRH2 cells, which express both *OCT4* and *SPP1* transcripts, was much higher than that of parental HCC827 cells (Fig. 3a and b). We then identified OCT4-positive cells using an OCT4-FLAG-tagged genomic transgene (pOCT4-Gen-FLAG) [16] containing CMV-RFP sequences to visualise transfected cells (illustrated in Fig. 3c). The ratio of cells that were stained for the FLAG antibody to those expressing RFP was higher in HCC827 GRH2 cells (53/800; 6·6%) than in parental HCC827 cells (1/184; < 0.5%) (Fig. 3c), indicating a higher percentage of OCT4-positive cells in HCC827 GRH2 cells relative to HCC827 cells, which is consistent with the endogenous transcript levels (Fig. 2c).

To clarify the role of OCT4-positive cancer cells in the highly aggressive HCC827 GRH2 cell line, we evaluated the effects of DTA-mediated ablation. We transfected the cells with either pOCT4-DTA or pOCT4-GFP (Fig. 3d) and observed that the cellular morphology was not significantly altered by pOCT4-DTA (Fig. 3d); however, the percentage of OCT4-positive cells dropped from 5.9 to 1.1% (Fig. 3e). Moreover, sphere-formation was not significantly affected by ablating OCT4-positive cells from the HCC827 GRH2 culture (Fig. 3f), but the migration significantly decreased (Fig. 3g). These data indicated that the OCT4-positive subpopulation might enhance HCC827 GRH2 cell migration, similar to its effect on the poorly differentiated endometrial cancer cell line HEC50B [16]. Furthermore, ablating OCT4-positive cells caused a significant decrease in *SPP1C* mRNA levels (Fig. 3h), suggesting that OCT4 and SPP1C might be expressed by the same cancer cells. These data strongly suggested that the OCT4/SPP1 axis might enhance the aggressiveness of somatic cancers. Therefore, we evaluated the potential significance of *OCT4-* and *SPP1*-transcript variants in human clinical tumour tissues.

### The OCT4/SPP1 axis in early-stage LUAD patients

**Fig. 4:**
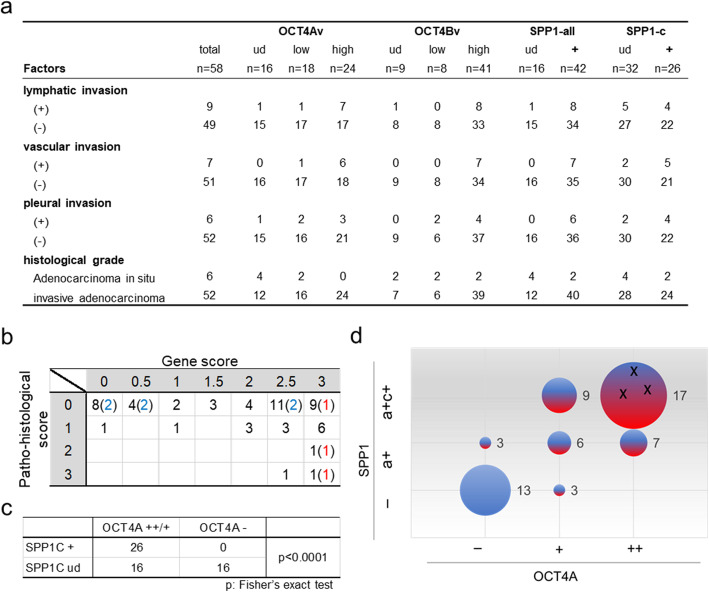
The OCT4/SPP1 axis in early-stage LUAD. **a** Summary of pathohistological factors, as well as *OCT4-* and *SPP1*-transcript expression in tumours from 58 LUAD patients. Values represent the number of patients harbouring tumours with designated characteristics. **b** Relationship between gene expression in the OCT4A/SPP1 axis and pathohistological scores. OCT4A scores: not detected (0), low levels (1), and high levels (2); SPP1 scores: not detected (0), *SPP1-*all detected (0.5), and both *SPP1*-all and *SPP1C* detected (1). Gene scores represent the total scores of OCT4A and SPP1. Criteria for evaluation of their expression levels was shown in Fig. S3. The pathohistological score is indicated as a total of each pathological score: Lymphatic invasion (Ly) (+): 1; vascular (Ve) (+): 1; and pleural invasion (pl) (+): 1. Blue numbers indicate cases of adenocarcinoma in situ, and red numbers indicate relapse cases. **c** Correlation between *OCT4A* and *SPP1C* expression in tumour tissues. The X-axis indicates *SPP1C*-expression levels (detected or undetected), and the Y-axis indicates *OCT4A*-expression levels (high and low levels or undetected). **d** Scatter plot showing the relationship between gene expression related to the OCT4A/SPP1 axis and levels of pathohistological risk. Each scatter point is represented by the following: an X-coordinate for *OCT4A*-expression levels (−: undetected; Low: detected at low levels; and High: detected at high levels), a Y-coordinate for *SPP1*-all and/or *SPP1C* expression (−: undetected; a: detection of *SPP1*-all detected; a + c: detection of *SPP1*-all and *SPP1C*); plot size: case number (each number indicates the number of cases); and colour [each colour represents pathohistological risk (blue: negative; and red: positive)]. Three black “X”s indicate that all three patients relapsed or had distant metastases during the observation period (case nos. 16, 59, and 62; Table S5)

LUAD is the most common subtype of lung cancer, which is a major global health threat owing to its high disease-associated mortality rates [31]. To examine whether the OCT4/SPP1 axis can help identify patients with early-stage LUAD at high-risk of metastasis, we investigated primary tumours of early-stage (Stage I) LUAD patients (Table S5), which were collected from surgical patients at Okayama University. Using the RT-PCR method described above, we identified the *OCT4-* and *SPP1*-transcript variants expressed in primary tumour samples of 58 patients and compared them with the histological grade and tissue-specific invasion data (Fig. 4 and Fig. S3; Table S5). We separated expression levels into two or three categories. *OCT4A* and *OCT4Bv*: high, easily detectable; low, faintly detectable; and minus, undetectable. *SPP1*-all: detectable (d) or undetectable (ud). SPP1C: clear bands, reproducibly detectable (d); or very few or no bands, undetectable (ud) (Fig. S3).

*OCT4Bv* was detected in 49 of the 58 clinical tumour samples, 42 of these also expressing *OCT4A* transcripts (Fig. 4a and Table S5). These results suggest that OCT4A and OCT4Bv might play a significant role in the development and progression of human LUAD. Within individual tumours, *OCT4A-*expression levels might correlate with the number of OCT4-positive cells, which are characterised by enhanced migration in lung cancer cell lines (Fig. 2e and Fig. 3g). All tumour samples expressing *OCT4A* at high levels also expressed high levels of *OCT4Bv* (24/24; Table S5). Assessing accurate *OCT4A* expression levels in tumour tissues might help diagnose LUAD. Alternatively, *SPP1* transcripts were detected in multiple tumour samples (42/58) (Fig. 4a), consistent with previous reports [38, 39]. However, *SPP1C* transcript variants were detected in < 50% the samples (26/58; Fig. 4a and Table S5), suggesting that *SPP1C* expression might be a potential diagnostic and prognostic marker for early-stage LUAD, which is consistent with previous studies of breast and ovarian cancers [37, 40]. However, we found that all tumours expressing *SPP1C* also expressed *SPP1A* and/or *SPP1B* (Table S5); therefore, our data do not refute a potential role for SPP1A and/or SPP1B isoforms in LUAD tumours. Nevertheless, *SPP1C* expression, which closely correlates with the overexpression of full-length *SPP1*, might represent a diagnostic marker for early-stage LUAD.

Histopathological assessment is indispensable for accurate diagnosis of lung cancer. Local vascular invasion is closely associated with an increased risk of future metastasis and death after surgery for early-stage NSCLC [41]; therefore, we examined the potential relationship between the OCT4A/SPP1C axis and local micro invasion in early-stage LUAD tumours (Fig. 4a). The lymphatic invasion was found in nine patients. Of these nine tumours, *OCT4A* was detected in eight, and *SPP1C* was detected in four. Similarly, vascular invasion was found in seven patients. Of these, all seven tumours expressed *OCT4A,* and five expressed *SPP1C*. Pleural invasion was found in six patients, all of which expressed *OCT4A,* and five expressed *SPP1C*. In patients with histopathological local invasion (score 1–3), *OCT4A* and/or *SPP1C* expression was detected in most tumours (16/17; 94%), *OCT4A* and *SPP1C* co-expression was detected in 10/17 cases (59%), only *OCT4A* expression in 5/17 cases (29%), and no tumours expressed only *SPP1C* (0/17; 0%). To evaluate the relationship between the OCT4/SPP1C axis and the presence of local invasion, we scored and compared the results (Fig. 4b and c). Remarkably, almost no cases showed local invasion in which the tumours expressed no *OCT4* or *SPP1* transcripts (gene score: 0; 1/9). Alternatively, in the six tumours lacking micro invasion, categorised as a subtype of adenocarcinoma in situ (Fig. 4b, blue numbers), a range of *OCT4* and *SPP1* gene scores were observed. Similarly, in cases with no available pathohistological observations, the gene scores were variable (Fig. 4b, pathohistological score: 0). Further investigation on the outcomes of these patients will clarify whether the OCT4A/SPP1C axis could reflect postoperative prognoses for patients without any pathohistological risk.

Notably, the tumours of all three patients who relapsed or had distant metastases during our observation period belonged to the group with the highest gene score [Figs. 4b and d; and Table S5 (case numbers 16, 59, and 62)]. Among these patients, one (No. 16) had been diagnosed with no pathohistological score (Fig. 4b and Table S5). These data might suggest that higher gene scores are associated with poorer outcomes, regardless of pathohistological features; however, additional cases must be examined to confirm our hypothesis. Additionally, we detected *OCT4* and *SPP1* transcripts at high frequencies in tumours with invasive regions (gene score: ≥2.0; 15/17) (Fig. 4b and Table S5). Fig. 4c and d show a significant correlation between *OCT4A* and *SPP1C* expression in LUAD, according to Fisher’s exact test. Interestingly, no tumours were *OCT4A* (−)/*SPP1C* (+), suggesting that OCT4A might directly or indirectly up-regulate *SPP1C* expression, which is consistent with the data shown in Fig. 3h. Conversely, it is possible that *OCT4A* (−)/*SPP1C* (−) status might predict good clinical outcomes (Fig. 4d; *n* = 13). We proffer that these data suggest that tumours expressing *OCT4A* and *SPP1C* might have different cells of origin and display different pathological phenotypes than *OCT4A* (−)/*SPP1C* (−) tumours.

## Discussion

This represents the first study reporting conclusive evidence of *OCT4*-transcript variants in healthy and cancerous human tissues. Remarkably, we readily detected *OCT4A* and *OCT4B* variants in some adult human somatic tissues, as well as in the gonads (Fig. 1). In contrast, none of the *Oct4* transcripts was detected in any of the examined tissues except the gonads from 4-month-old mice (Fig. S1). We believe that these unexpected results will be of value to oncology researchers and those investigating stem cell biology. Most researchers routinely regard the mouse as a suitable model for humans, and thus, results obtained using mice are frequently applied to humans without adequate consideration. Our data (Fig. 1) should rekindle interest in the physiological and/or pathological functions of OCT4-expressing cells in adulthood.

Until now, the somatic function of OCT4 remained unclear. We previously reported that OCT4A and B164 (OCT4C) isoforms might be the major products translated from *OCT4A* and *OCT4Bv* mRNAs [16]. It was predicted that the OCT4C protein isoform lacking the N-terminal portion of OCT4A would not function as a transcription factor (OCT4A) despite its ability to transform fibroblasts in vitro, similar to OCT4A [16]. The different expression patterns of *OCT4A* and *OCT4Bv* (Fig. 1a) and the possible functional heterogeneity of OCT4 isoforms suggest that variant-specific detection is important to elucidate the function of human OCT4 in adult somatic tissues.

It remains unclear which cells in adult human somatic tissues express OCT4A. No *OCT4A*-transcripts were detected in bone marrow, MSC-AC, or MCS-BM (Fig. 1a), suggesting that *OCT4A*-expressing somatic cells are not hematopoietic or mesenchymal stem cells. Moreover, *OCT4A* expression in human somatic tissues appeared lower than that of PA-1, undifferentiated ovarian teratocarcinoma cells, although levels in the lung tissue were higher than the testis (Fig. 1d). If OCT4A-positive somatic cells are scarce, it is likely that those expressing *OCT4A* do so at significantly higher levels in certain somatic tissues. These may be TICs or CSCs, and like those in germ-cell tumours [6, 10], they may give rise to certain cancers in human somatic tissues. To characterise the physiological and pathological roles of OCT4 in human somatic cells, we could use, after appropriate genetic modification, animal model other than mice as a new model for humans.

In our previous and current analyses, we identified subpopulations of OCT4-positive cells in the lung and endometrial adenocarcinoma cell lines [16] that promoted cell migration and invasion (Fig. 2e and Fig. 3g). High expression levels of *OCT4A* and *OCT4Bv*, suggesting that OCT4-positive cells are relatively common among cancer cell lines and tumours, were closely related to *SPP1* expression, and ablation of OCT4-positive cells significantly decreased *SPP1C*-expression levels, suggesting that OCT4A might directly or indirectly up-regulate *SPP1*C expression in a subpopulation of cancer cells. Additionally, we confirmed a significant correlation between *OCT4A* and *SPP1C* expression in primary LUAD tumours (Fig. 4c). Numerous studies suggest the importance of *SPP1*-transcript variant expression in cancer progression and prognosis [42, 43]. The results of the present study suggest that cancer cells positive for the OCT4A/SPP1C axis might promote the aggressiveness of a variety of cancers.

Lung cancer is a major global health threat associated with high disease-associated mortality rates [31]. The overall prognosis for patients with lung cancer is poor, and even for patients with early-stage disease, the postoperative recurrence rate is high relative to other cancers. The present study strongly suggests that co-expression of *OCT4A* and *SPP1C* transcript variants may represent a prognostic marker and therapeutic target for stage I LUAD, and that their products may play a significant role in LUAD development and progression. Limitations of our study include the small number of patients enrolled and the short follow-up period. For further validation, larger-scale studies over longer timelines must be performed. A more convenient and quantitative method for the detection of *OCT4A-* and *SPP1C*-transcript variants might also be required. We suggest that the variant-specific transcripts analysis can be used more effectively than protein analysis for clinical applications, because the specificity of the OCT4A antibody remains an unsolved problem. Moreover, the molecular mechanisms responsible for the correlation between OCT4 and SPP1 in cancer cells was not elucidated; therefore, future work should focus on investigating the roles of OCT4A and SPP1C in tumour cell migration, as well as in other biological activities.

## Conclusion

We demonstrated the significance of *OCT4A-* and *SPP1C*-transcript variants in human carcinoma cell lines and clinical LUAD tumour tissues. Notably, our approach for identifying full-length splice variants highlights a “blind spot” in previous large-scale analyses. Our findings provide new insights into the basis of cell plasticity in normal and tumour tissues, which are essential for the design of more efficient therapies that selectively target CSCs/TICs [1, 2, 44]. The results of past studies must be reassessed, and expression data for *OCT4A-* and *SPP1C*-transcript variants should be collected using both bioinformatics analysis and the reliable methods described in this study. Our data suggest OCT4A as a potential marker for CSCs/TICs, at least in certain human epithelial tumours. Moreover, we hypothesise that up-regulated expression of *OCT4A* and *SPP1C* in cancer cells, especially during the early stages of malignant transformation, might indicate a propensity toward increased migration, aggressiveness, and progression of somatic cell tumours. We hope our findings help improve the accurate diagnosis and prediction of early-stage LUAD in a new era of personalised medicine and provide a new approach for targeting tumour cell motility to prevent metastasis.

## Supplementary information

**Additional file 1: Table S1.** Cell lines used in this study, **Table S2.** RNA resources used for analysis, **Table S3.** PCR primer sets for RT-PCR and qPCR, **Table S4.** Analysis of DNA variations according to sequencing analysis, **Table S5.** Summary of clinical data and OCT4/SPP1 expression analysis.

**Additional file 2: Fig. S1.** Oct4a and Oct4b transcript variants are not expressed in adult murine somatic tissues, **Fig. S2** Expression of SPP1C in normal human adult tissues, **Fig. S3** Criteria for evaluation of OCT4/SPP1 transcript expression analysis for clinical tumour samples, **Fig. S4**–1 Uncropped full-length gel images related to Fig. 1a (hOCT4A), **Fig. S4**–2 Uncropped full-length gel images related to Fig. 1a (OCT4Bv), **Fig. S4**–3 Uncropped full-length gel images related to Fig. 1a (GAPDH), **Fig. S4**–4 Uncropped full-length gel images related to Fig. 1c, **Fig. S5**–1 Uncropped full-length gel images related to Fig. 2a, **Fig. S5**–2 Uncropped full-length gel images related to Fig. 2b, **Fig. S5**–3 Uncropped full-length gel images related to Fig. 2c (OCT4A, OCT4Bv), **Fig. S5**–4 Uncropped full-length gel images related to Fig. 2c (SPP1all, SPP1C), **Fig. S5**–5 Uncropped full-length gel images related to Fig. 2c (GAPDH), **Fig. S6**–1 Uncropped full-length gel images related to **Fig. S1**a, **Fig. S6**–2 Uncropped full-length gel images related to **Fig. S1**b, **Fig. S7** Uncropped full-length gel images related to **Fig. S2** (SPP1C), **Fig. S8**–1 Uncropped full-length gel images related to **Fig. S3** (OCT4A, OCT4Bv), **Fig. S8**–2 Uncropped full-length gel images related to **Fig. S3** (SPP1all, SPP1C), **Fig. S8**–3 Uncropped full-length gel images related to **Fig. S3** (GAPDH)

## Data Availability

All data generated in the study are included in this article. The data that support the findings of this study are available from the corresponding author upon reasonable request.

## Abbreviations

OCT4: Octamer-binding transcription factor
SPP1: Secreted phosphoprotein 1
CSCs: Cancer stem cells
TICs: Tumour-initiating cells
RT-PCR: Reverse transcription polymerase chain reaction
qPCR: Real-time quantitative PCR
LUAD: Lung adenocarcinoma
DT-A: Diphtheria toxin fragment A
SD: Standard deviation
GAPDH: Glyceraldehyde 3-phosphate dehydrogenase
OCT4Bv: OCT4B splice variants
