# Supplementary material for: Prognostic value of OCT4A and SPP1C transcript variant co-expression in early-stage lung adenocarcinoma
Source: BMC Cancer. 2020 Jun 5;20:521. doi: 10.1186/s12885-020-06969-0 (PMC7275395; doi:10.1186/s12885-020-06969-0)

**Supplemental Tables S1-S5**

| Table S1 Cell lines used in this study | |
| --- | --- |
| Cell line | Cell type |
| *Carcinoma* |  |
| PA-1 | Human ovary teratocarcinoma cell line |
| MCF7 | Human breast adenocarcinoma (well-differentiated) cell line |
| MDAMB231 | Human breast adenocarcinoma (poorly differentiated) cell line |
| Ishikawa | Human endometrial adenocarcinoma (well-differentiated) cell line |
| HEC50B | Human endometrial adenocarcinoma (poorly differentiated) cell line |
| HCC827 | Human lung adenocarcinoma (parental) cell line |
| HCC827 GRH2 | Human lung adenocarcinoma (gefitinib-resistant) cell line |
| HCC827 ARH | Human lung adenocarcinoma (afatinib‐resistant) cell line |
| A549 | Human lung adenocarcinoma cell line |
| H1299 | Human lung large cell carcinoma cell line |
| PC-9 | Human lung adenocarcinoma (well-differentiated) cell line |
| HCC4006 | Human lung adenocarcinoma cell line |
| H1975 | Human lung adenocarcinoma cell line |
| *Mesothelioma* |  |
| MSTO-211H | Human malignant biphasic mesothelioma cell line |
| H2052 | Human malignant mesothlioma cell line |
| *Non-malignant cells* |  |
| HBEC-5KT | Human immortalized bronchial epithelial cell line |
| MeT-5A | Human immortalized mesothelial cell line |

HCC827 GRH2 and HCC827 ARH had been established from parental cell lines HCC827 in a prior study.32 HBEC-5KT is a non-malignant bronchial epithelial cell line established by expressing hTERT and Cdk4 genes (Cancer Res. 2004;64:9027-34). Met-5A is a TERT-transformed normal mesothelial cell line (Am. J Pathol. 1989;134:979-91)

| Table S2 RNA resources used for analysis | | |  |
| --- | --- | --- | --- |
|  | Sex | Age |  |
| Human |  |  |  |
| Whole eye | female | 37 weeks | BioChain, catalog number R1244108-10 |
| Thyroid | male | 50 years | Agilent Technologies, catalog number 540039 |
| Lung | male | 34 and 59 years (pooled from 4 donors) | Agilent Technologies, catalog number 540019 |
| Ovary | female | 47 years | Agilent Technologies, catalog number 540071 |
| Testis | male | 21 - 29 years (pooled from 5 donors) | Clontech, catalog number 636533 |
| Uterus | female | 23 - 63 years (pooled from 8 donors) | Clontech, catalog number 636551 |
| Pancreas | female | 76 years | Agilent Technologies, catalog number 540023 |
| Breast | female | 40 - 55 years (pooled from 5 donors) | Agilent Technologies, catalog number 540045 |
| Bone marrow | male/female | 58 - 76 years (pooled from 4 donors) | Clontech, catalog number 636643 |
| Peripheral leukocyte | male/female | 18 - 54 years (pooled from 426 donors) | Clontech, catalog number 636592 |
| Heart | male | 30, 30 and 39 years (pooled from 3 donors) | Clontech, catalog number 636643 |
| Cerebellum | male/female | 16 - 70 years (pooled from 24 donors) | Clontech, catalog number 636643 |
| Kidney | female | 40 years | Clontech, catalog number 636643 |
| Liver | male/female | 24 - 64 years (pooled from 3 donors) | Clontech, catalog number 636643 |
| Placenta | female | 23 - 30 years (pooled from 3 donors) | Clontech, catalog number 636643 |
| Skeletal muscle | male | 20 years | Clontech, catalog number 636643 |
| Spleen | male/female | 22 - 69 years (pooled from 15 donors) | Clontech, catalog number 636643 |
| Colon | male | 23 - 63 years (pooled from 3 donors) | Clontech, catalog number 636643 |
| Small intestine | male/female | 20 - 61 years (pooled from 5 donors) | Clontech, catalog number 636643 |
| Spinal code | male | 20 - 59 years (pooled from 7 donors) | Clontech, catalog number 636643 |
| Stomach | male | 40 years | Clontech, catalog number 636643 |
| Lung | male | 55 years | Zyagen, catalog number HR-601 |
| Lung | male | 23 years | BioChain, catalog number R1234152-50 |
| MSCs from adipose tissue (Msc-AT) | |  | PromoCell catalog number C-14092 |
| MSCs from bone marrow (Msc-BM) | |  | PromoCell catalog number C-14090 |
| Mouse |  |  |  |
| Testis | male | 4 months (pooled from 2 animals) | Japan SLC, C57BL/6N |
| Ovary | female | 4 months (pooled from 2 animals) | Japan SLC, C57BL/6N |
| Somatic tissues | male/female | 4 months (pooled from 4 animals) | Japan SLC, C57BL/6N |
| Postnatal eye | male/female | 3 days (independent 4 animals) | Japan SLC, C57BL/6N |

| Table S3 PCR primer sets for RT-PCR and qPCR | | | | |
| --- | --- | --- | --- | --- |
|  | Primer | | Sequence | Product size |
| Human |  |  |  |  |
| OCT4A | forward | hOCT4A-FO | 5′-AGAGAGGGGTTGAGTAGTCCCTTCGCA-3′ | 1347(A), 1465(A1) bp |
|  | reverse | hOCT4A, B-RV1 | 5′-CAAGAGCATCATTGAACTTCACCTTC-3′ |  |
| OCT4Bv | forward | hOCT4B-FO | 5′-AGGCAGATGCACTTCTACAGACTATTC-3′ | 995(B), 1228(B1),  1512(B2), 1774 (Bns) bp |
|  | reverse | hOCT4A, B-RV1 | 5′-CAAGAGCATCATTGAACTTCACCTTC-3′ |  |
| GAPDH | forward | hGAPDH-FO | 5′-GCTTGTCATCAATGGAAATCCC-3′ | 210 bp |
|  | reverse | hGAPDH-RV | 5′-TTCACACCCATGACGAACATG-3′ |  |
| Mouse |  |  |  |  |
| Oct-3/4A | forward | mOct-3/4A-FO | 5′-CCCCAATGCCGTGAAGTTGGAGAAGGT-3′ | 452 bp |
|  | reverse | mOct-3/4A, B-RV | 5′-TCTCTAGCCCAAGCTGATTGGCGATGTG-3′ |  |
| Oct-3/4B | forward | mOct-3/4B-FO1 | 5′-GACTCTGACAAGTCTGCCTTTCTCACT-3′ | 437 bp |
|  | forward | mOct-3/4B-FO2 | 5′-AGCCTTAAAACTTCTTCAGAATAGGTC-3′ | 430 bp |
|  | forward | mOct-3/4B-FO3 | 5′-ATGAAAGCCCTGCAGAAGGAGCTAGAACA-3′ | 397 bp |
|  | reverse | mOct-3/4A, B-RV | 5′-TCTCTAGCCCAAGCTGATTGGCGATGTG-3′ |  |
| G3pdh | forward | mGAPDH-FO | 5′-CTGTGGTACAAGAGGTGGACACA-3′ | 308 bp |
|  | reverse | mGAPDH-RV | 5′-GCATCTCCTTCTCCAGCTGCTCA-3′ |  |
| Human |  |  |  |  |
| SPP1all | forward | hSPP1all-FO | 5′-ACCATGAGAATTGCAGTGATTTGC-3′ | 407(a), 365(b), 327(c) bp |
|  | reverse | hSPP1all, c-RV | 5′-TCAGTGACCAGTTCATCAGATTCA-3′ |  |
| SPP1C | forward | hSPP1c-FO | 5′-CTGGAAGTTCTGAGGAAAAGCAGAATG-3′ | 253 bp |
|  | reverse | hSPP1all, c-RV | 5′-TCAGTGACCAGTTCATCAGATTCA-3′ |  |
| 18S rRNA | forward | 18S-FO | 5′-ACCCGTTGAACCCCATTCGTGA-3′ | 159 bp |
|  | reverse | 18S-RV | 5′-GCCTCACTAAACCATCCAATCGG-3′ |  |
| beta-ACTIN | forward | ACTB-FO | 5′-CACCATTGGCAATGAGCGGTTC-3′ | 135 bp |
|  | reverse | ACTB-RV | 5′-AGGTCTTTGCGGATGTCCACGT-3′ |  |
| SPP1 all: a common primer pair for detection of SPP1A, SPP1B and SPP1C | | | |  |

| Table S4 Analysis of DNA variations according to sequencing analysis | | | | | | | | |
| --- | --- | --- | --- | --- | --- | --- | --- | --- |
|  | OCT4A clone # | | | OCT4B clone # | | | | |
|  | A | A1 | total | B | B1 | B2 | Bns | total |
| Eye | 7 | 1 | 8 | 11 | 3 | 0 | 2 | 16 |
| Pancreas | 7 | 0 | 7 | 9 | 0 | 2 | 1 | 12 |
| Thyroid | 6 | 0 | 6 | 5 | 0 | 0 | 7 | 12 |
| Testis | 7 | 0 | 7 | 6 | 2 | 1 | 0 | 9 |
| Uterus | 3 | 0 | 3 | 3 | 2 | 0 | 2 | 7 |
| Lung | 5 | 3 | 8 | 12 | 3 | 3 | 0 | 18 |
| Breast | 4 | 0 | 4 | 11 | 3 | 5 | 2 | 21 |
| OCT4, octamer-binding transcription factor 4 | | | | |  |  |  |  |


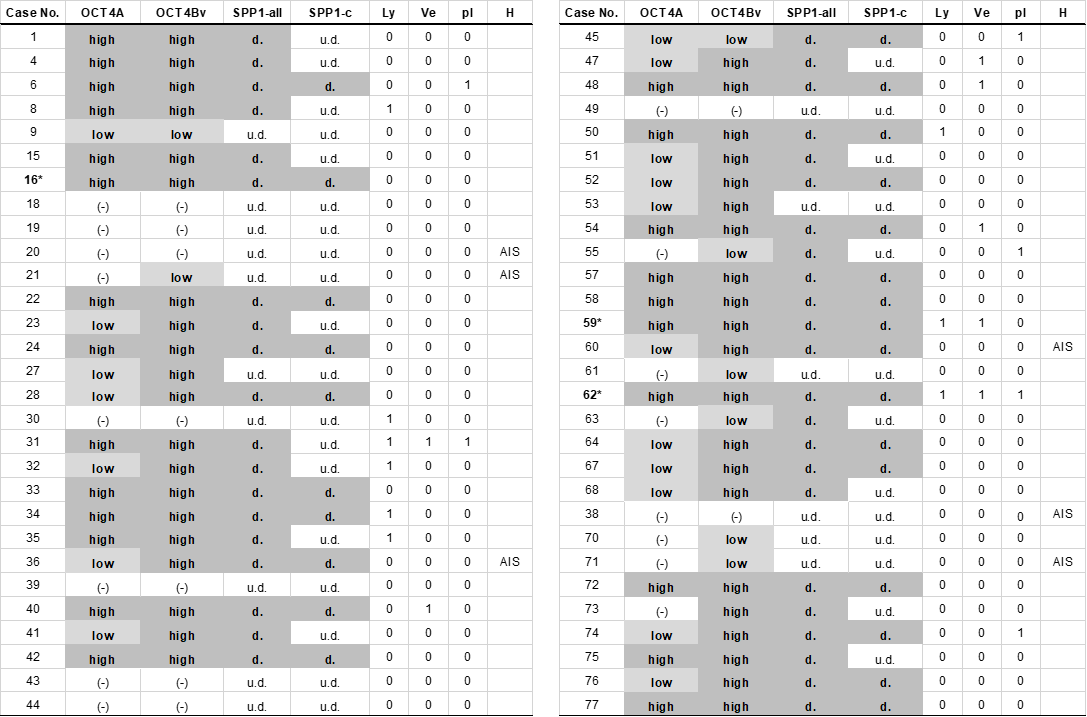
Table S5 Summary of clinical data and OCT4/SPP1 expression analysis


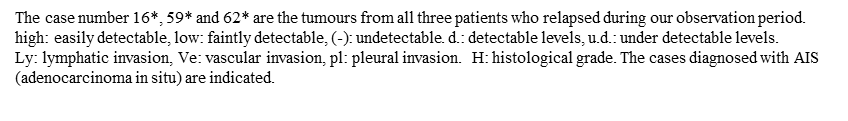

Supplement: Supplementary file 1 — Additional file 1: Table S1. Cell lines used in this study, Table S2. RNA resources used for analysis, Table S3. PCR primer sets for RT-PCR and qPCR, Table S4. Analysis of DNA variations according to sequencing analysis, Table S5. Summary of clinical data and OCT4/SPP1 expression analysis. [file 12885_2020_6969_MOESM1_ESM.docx]
