# Supplementary material for: Prognostic value of OCT4A and SPP1C transcript variant co-expression in early-stage lung adenocarcinoma
Source: BMC Cancer. 2020 Jun 5;20:521. doi: 10.1186/s12885-020-06969-0 (PMC7275395; doi:10.1186/s12885-020-06969-0)
Supplement: Supplementary file 2 — Additional file 2: Fig. S1. Oct4a and Oct4b transcript variants are not expressed in adult murine somatic tissues, Fig. S2 Expression of SPP1C in normal human adult tissues, Fig. S3 Criteria for evaluation of OCT4/SPP1 transcript expression analysis for clinical tumour samples, Fig. S4–1 Uncropped full-length gel images related to Fig. 1a (hOCT4A), Fig. S4–2 Uncropped full-length gel images related to Fig. 1a (OCT4Bv), Fig. S4–3 Uncropped full-length gel images related to Fig. 1a (GAPDH), Fig. S4–4 Uncropped full-length gel images related to Fig. 1c, Fig. S5–1 Uncropped full-length gel images related to Fig. 2a, Fig. S5–2 Uncropped full-length gel images related to Fig. 2b, Fig. S5–3 Uncropped full-length gel images related to Fig. 2c (OCT4A, OCT4Bv), Fig. S5–4 Uncropped full-length gel images related to Fig. 2c (SPP1all, SPP1C), Fig. S5–5 Uncropped full-length gel images related to Fig. 2c (GAPDH), Fig. S6–1 Uncropped full-length gel images related to Fig. S1a, Fig. S6–2 Uncropped full-length gel images related to Fig. S1b, Fig. S7 Uncropped full-length gel images related to Fig. S2 (SPP1C), Fig. S8–1 Uncropped full-length gel images related to Fig. S3 (OCT4A, OCT4Bv), Fig. S8–2 Uncropped full-length gel images related to Fig. S3 (SPP1all, SPP1C), Fig. S8–3 Uncropped full-length gel images related to Fig. S3 (GAPDH) [file 12885_2020_6969_MOESM2_ESM.pptx]

## Slide 1
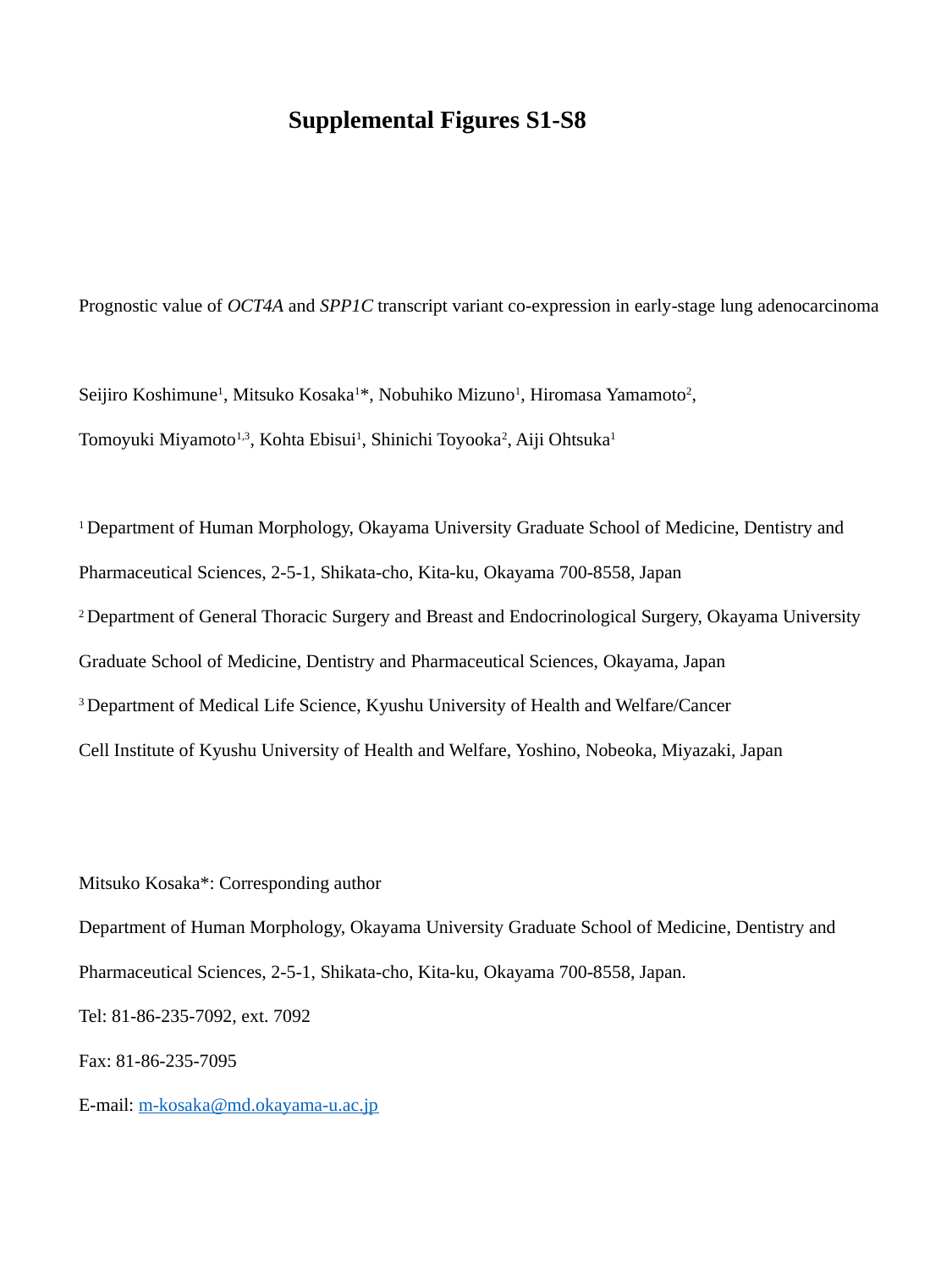

Supplemental Figures S1-S8
Prognostic value of OCT4A and SPP1C transcript variant co-expression in early-stage lung adenocarcinoma
Seijiro Koshimune1, Mitsuko Kosaka1*, Nobuhiko Mizuno1, Hiromasa Yamamoto2,
Tomoyuki Miyamoto1,3, Kohta Ebisui1, Shinichi Toyooka2, Aiji Ohtsuka1
1 Department of Human Morphology, Okayama University Graduate School of Medicine, Dentistry and Pharmaceutical Sciences, 2-5-1, Shikata-cho, Kita-ku, Okayama 700-8558, Japan
2 Department of General Thoracic Surgery and Breast and Endocrinological Surgery, Okayama University Graduate School of Medicine, Dentistry and Pharmaceutical Sciences, Okayama, Japan
3 Department of Medical Life Science, Kyushu University of Health and Welfare/Cancer
Cell Institute of Kyushu University of Health and Welfare, Yoshino, Nobeoka, Miyazaki, Japan
Mitsuko Kosaka*: Corresponding author
Department of Human Morphology, Okayama University Graduate School of Medicine, Dentistry and Pharmaceutical Sciences, 2-5-1, Shikata-cho, Kita-ku, Okayama 700-8558, Japan.
Tel: 81-86-235-7092, ext. 7092
Fax: 81-86-235-7095
E-mail: m-kosaka@md.okayama-u.ac.jp

## Slide 2
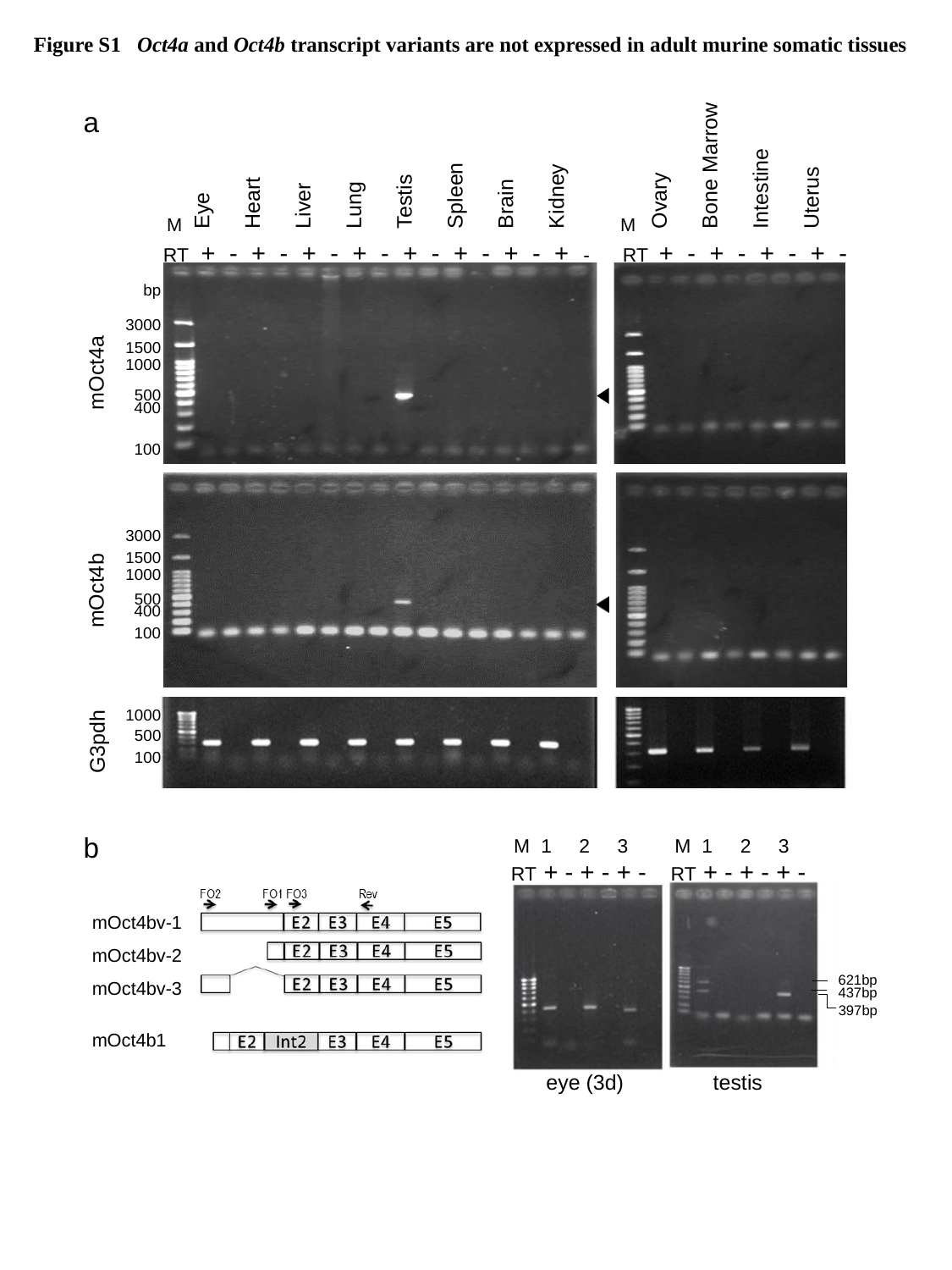

Figure S1 Oct4a and Oct4b transcript variants are not expressed in adult murine somatic tissues
a
Ovary
Bone Marrow
Intestine
Uterus
Eye
Heart
Liver
Lung
Testis
Spleen
Brain
Kidney
M
M
RT + - + - + - + - + - + - + - + - RT + - + - + - + -
bp
3000
1500
1000
mOct4a
500
400
100
3000
1500
1000
mOct4b
500
400
100
1000
 500
G3pdh
100
b
M 1 2 3
M 1 2 3
RT + - + - + -
RT + - + - + -
mOct4bv-1
mOct4bv-2
mOct4bv-3
mOct4b1
621bp
437bp
397bp
eye (3d)
 testis

## Slide 3
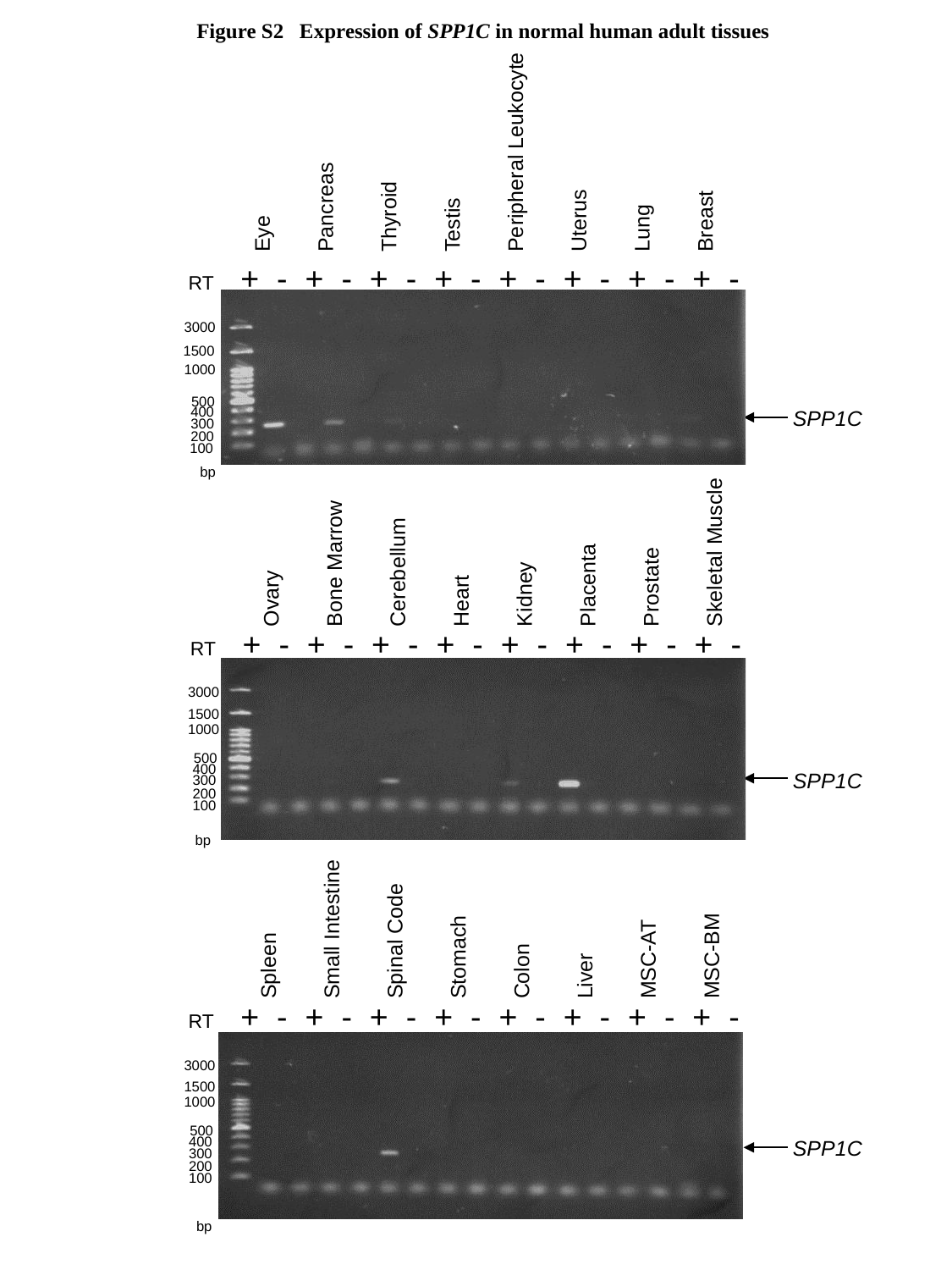

Figure S2 Expression of SPP1C in normal human adult tissues
Eye
Pancreas
Thyroid
Testis
Peripheral Leukocyte
Uterus
Lung
Breast
Ovary
Bone Marrow
Cerebellum
Heart
Kidney
Placenta
Prostate
Skeletal Muscle
RT + - + - + - + - + - + - + - + -
3000
1500
1000
500
400
SPP1C
300
200
100
bp
RT + - + - + - + - + - + - + - + -
3000
Spleen
Small Intestine
Spinal Code
Stomach
Colon
Liver
MSC-AT
MSC-BM
1500
1000
500
400
SPP1C
300
200
100
bp
RT + - + - + - + - + - + - + - + -
3000
1500
1000
500
400
SPP1C
300
200
100
bp

## Slide 4
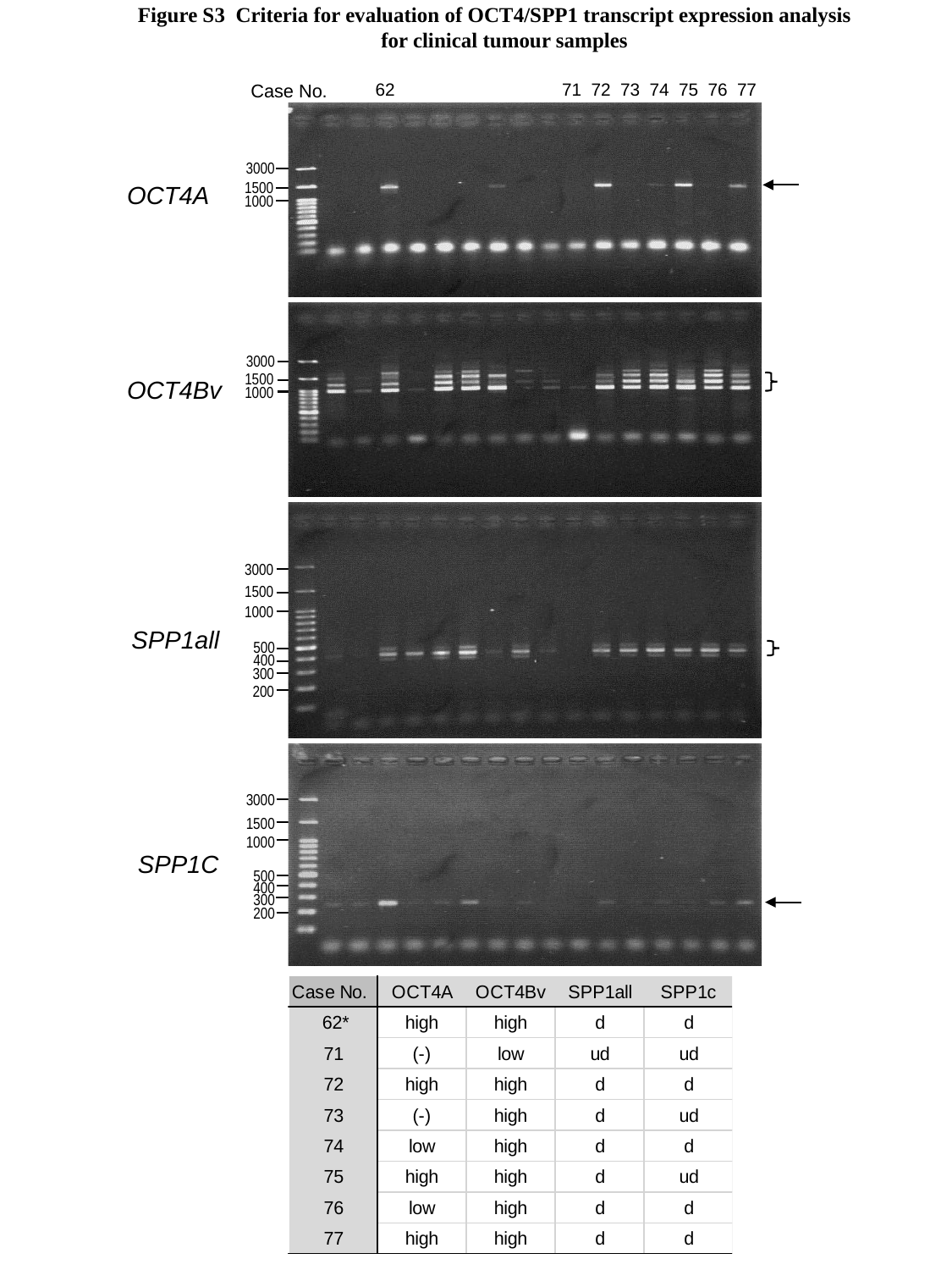

Figure S3 Criteria for evaluation of OCT4/SPP1 transcript expression analysis
 for clinical tumour samples
62
71 72 73 74 75 76 77
Case No.
3000
1500
OCT4A
1000
3000
1500
OCT4Bv
1000
3000
1500
1000
SPP1all
500
400
300
200
3000
1500
1000
SPP1C
500
400
300
200

## Slide 5
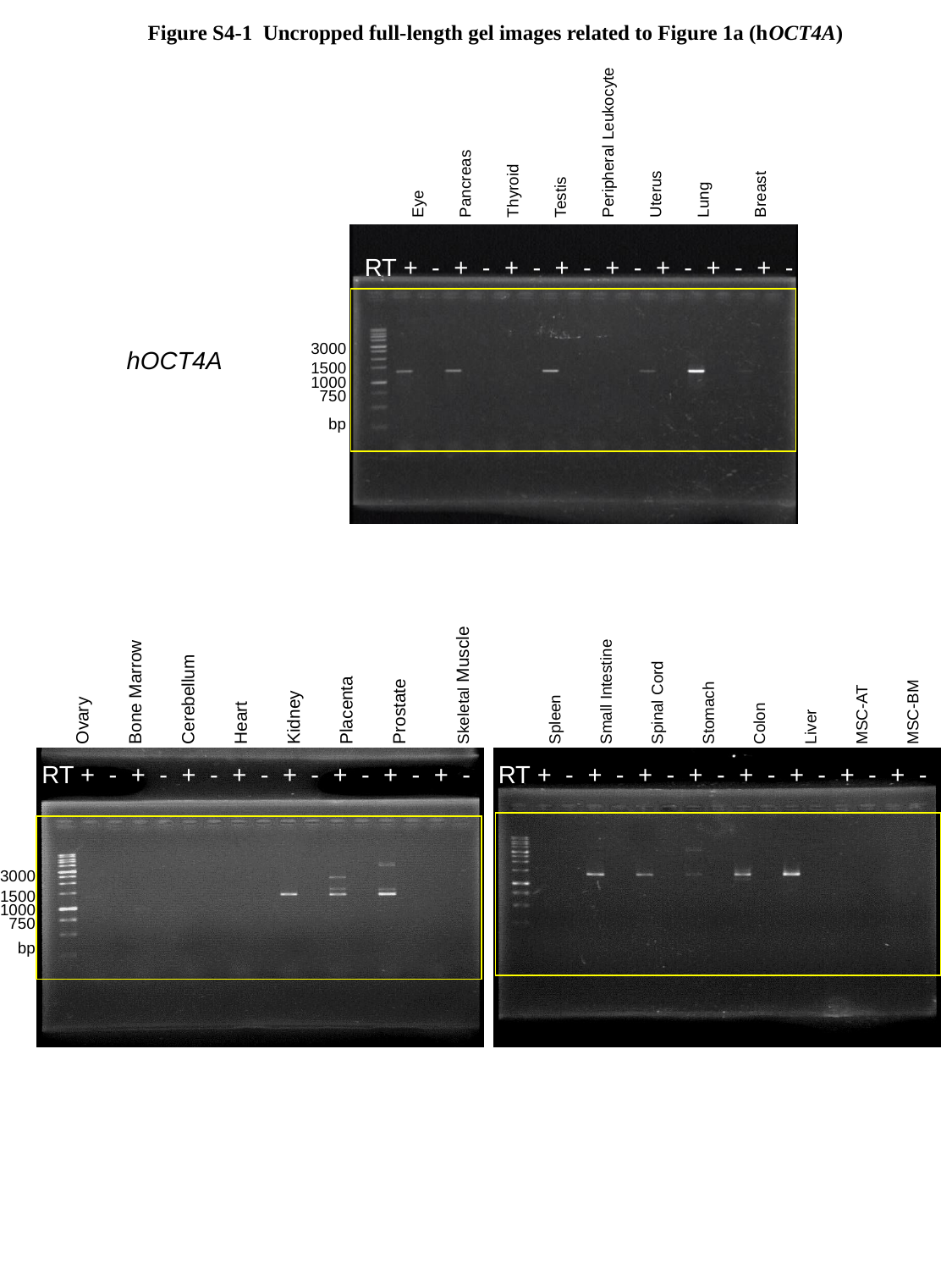

Figure S4-1 Uncropped full-length gel images related to Figure 1a (hOCT4A)
Eye
Pancreas
Thyroid
Testis
Peripheral Leukocyte
Uterus
Lung
Breast
RT + - + - + - + - + - + - + - + -
3000
hOCT4A
1500
1000
750
bp
Ovary
Bone Marrow
Cerebellum
Heart
Kidney
Placenta
Prostate
Skeletal Muscle
Spleen
Small Intestine
Spinal Cord
Stomach
Colon
Liver
MSC-AT
MSC-BM
RT + - + - + - + - + - + - + - + -
RT + - + - + - + - + - + - + - + -
3000
1500
1000
750
bp

## Slide 6
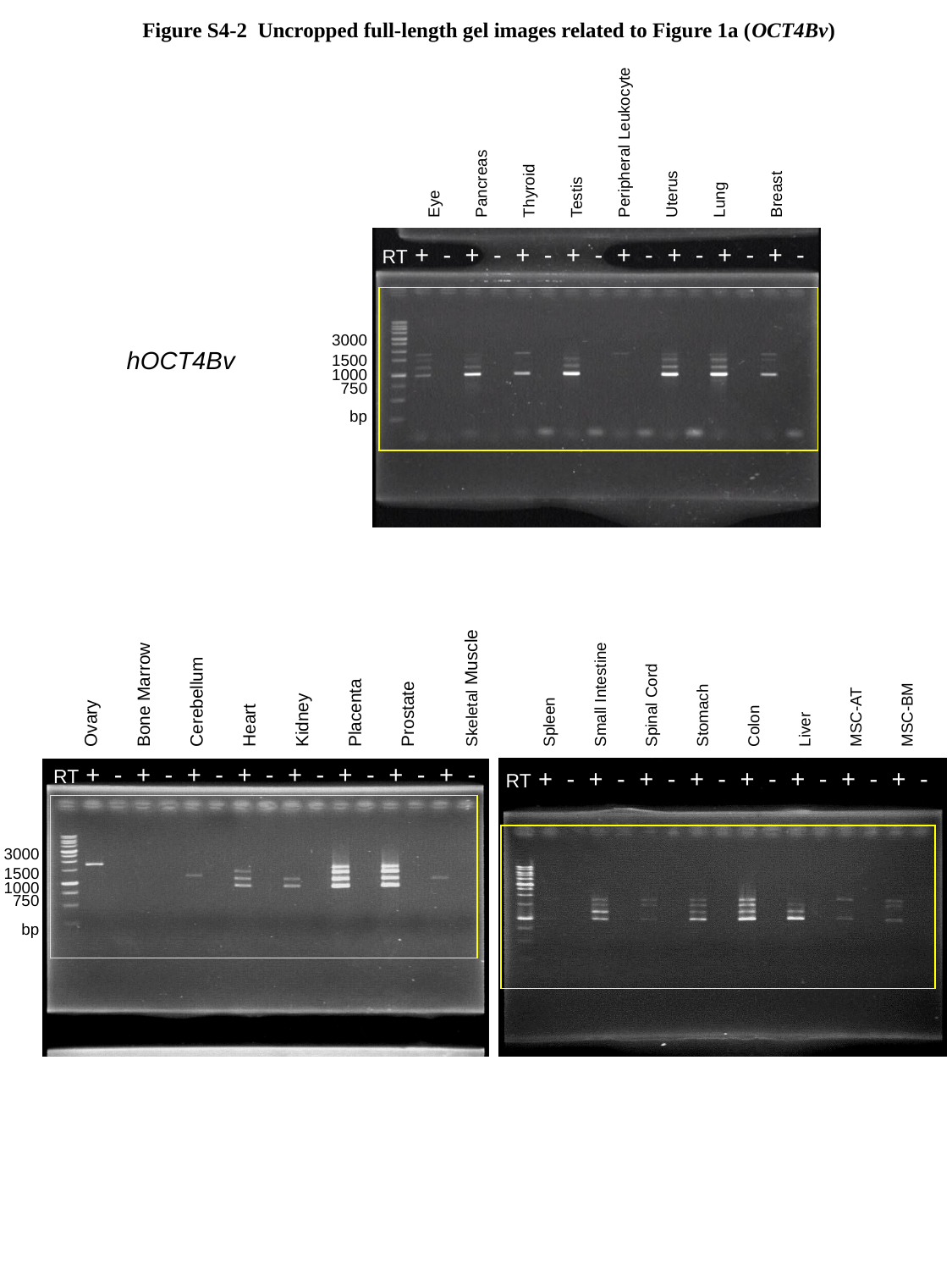

Figure S4-2 Uncropped full-length gel images related to Figure 1a (OCT4Bv)
Eye
Pancreas
Thyroid
Testis
Peripheral Leukocyte
Uterus
Lung
Breast
RT + - + - + - + - + - + - + - + -
3000
hOCT4Bv
1500
1000
750
bp
Ovary
Bone Marrow
Cerebellum
Heart
Kidney
Placenta
Prostate
Skeletal Muscle
Spleen
Small Intestine
Spinal Cord
Stomach
Colon
Liver
MSC-AT
MSC-BM
RT + - + - + - + - + - + - + - + -
RT + - + - + - + - + - + - + - + -
3000
1500
1000
750
bp

## Slide 7
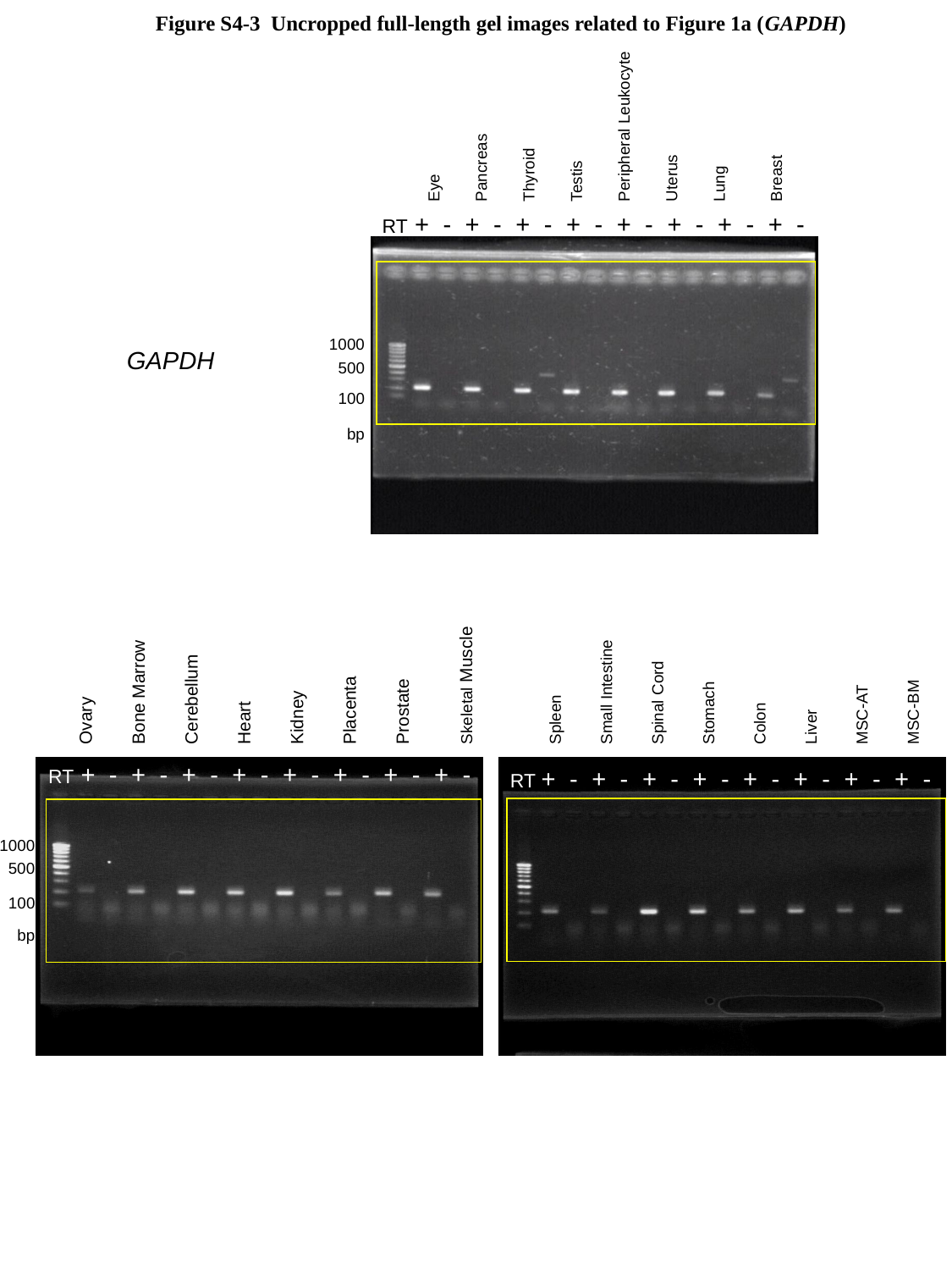

Figure S4-3 Uncropped full-length gel images related to Figure 1a (GAPDH)
Eye
Pancreas
Thyroid
Testis
Peripheral Leukocyte
Uterus
Lung
Breast
RT + - + - + - + - + - + - + - + -
1000
GAPDH
 500
100
bp
Ovary
Bone Marrow
Cerebellum
Heart
Kidney
Placenta
Prostate
Skeletal Muscle
Spleen
Small Intestine
Spinal Cord
Stomach
Colon
Liver
MSC-AT
MSC-BM
RT + - + - + - + - + - + - + - + -
RT + - + - + - + - + - + - + - + -
1000
 500
100
bp

## Slide 8
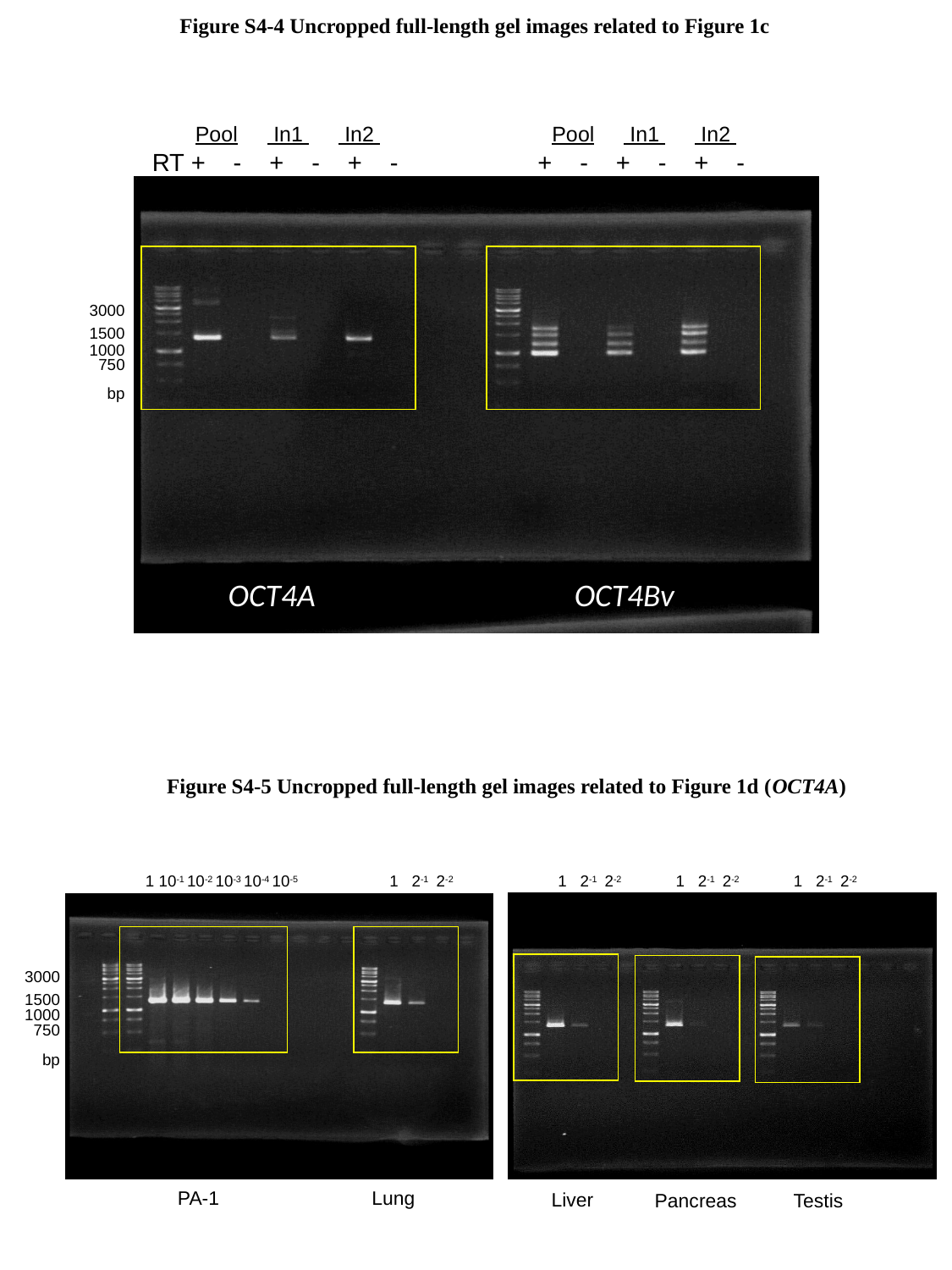

Figure S4-4 Uncropped full-length gel images related to Figure 1c
Pool In1 In2
Pool In1 In2
RT + - + - + - + - + - + -
3000
1500
1000
750
bp
OCT4A
OCT4Bv
Figure S4-5 Uncropped full-length gel images related to Figure 1d (OCT4A)
 1 10-1 10-2 10-3 10-4 10-5
1 2-1 2-2
1 2-1 2-2
1 2-1 2-2
1 2-1 2-2
3000
1500
1000
750
bp
PA-1
Lung
Liver
Pancreas
Testis

## Slide 9
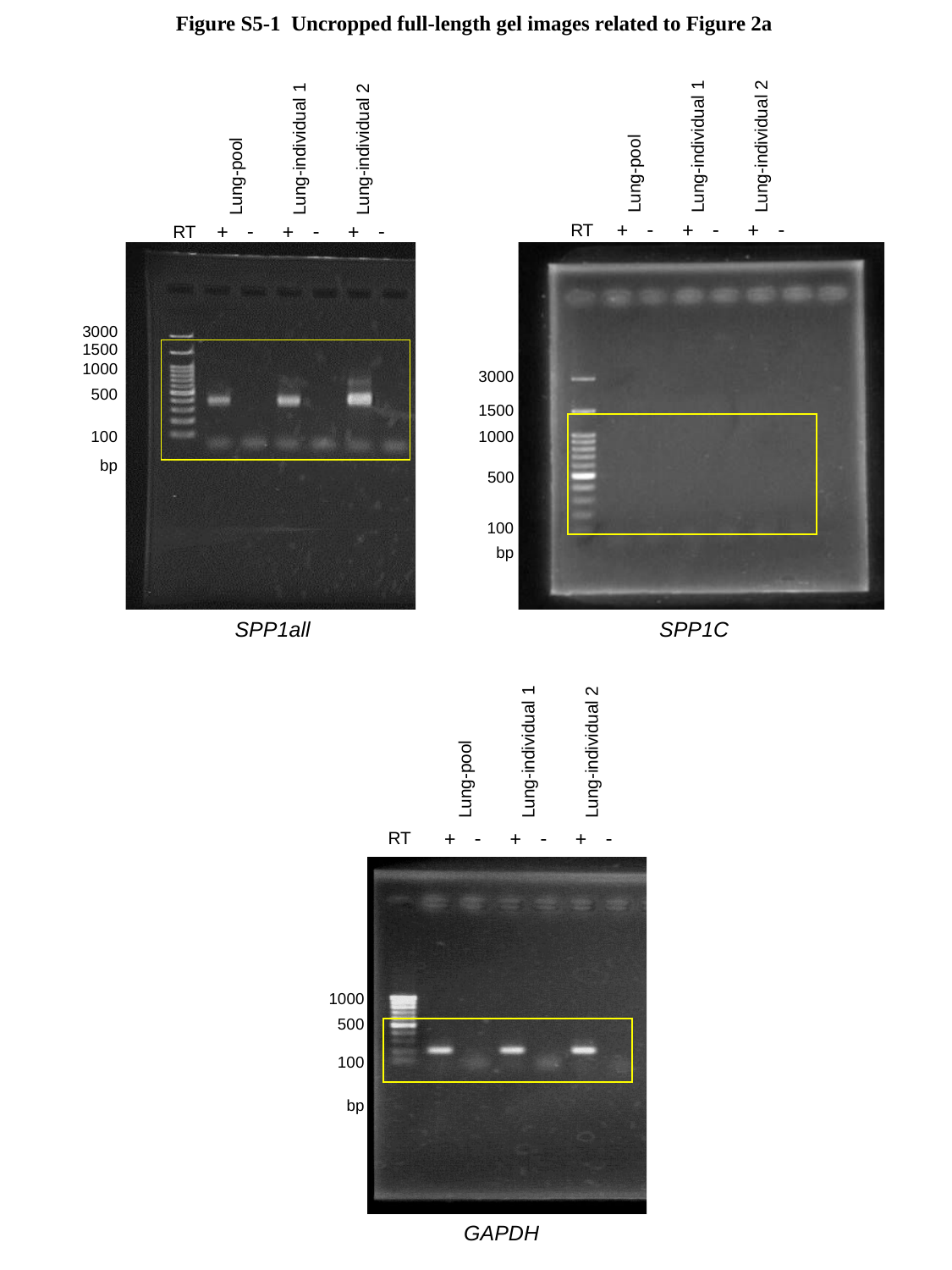

Figure S5-1 Uncropped full-length gel images related to Figure 2a
Lung-pool
Lung-individual 1
Lung-individual 2
Lung-pool
Lung-individual 1
Lung-individual 2
+ - + - + -
+ - + - + -
RT
RT
3000
1500
1000
3000
500
1500
100
1000
bp
500
100
bp
SPP1all
SPP1C
Lung-pool
Lung-individual 1
Lung-individual 2
+ - + - + -
RT
1000
 500
 100
bp
GAPDH

## Slide 10
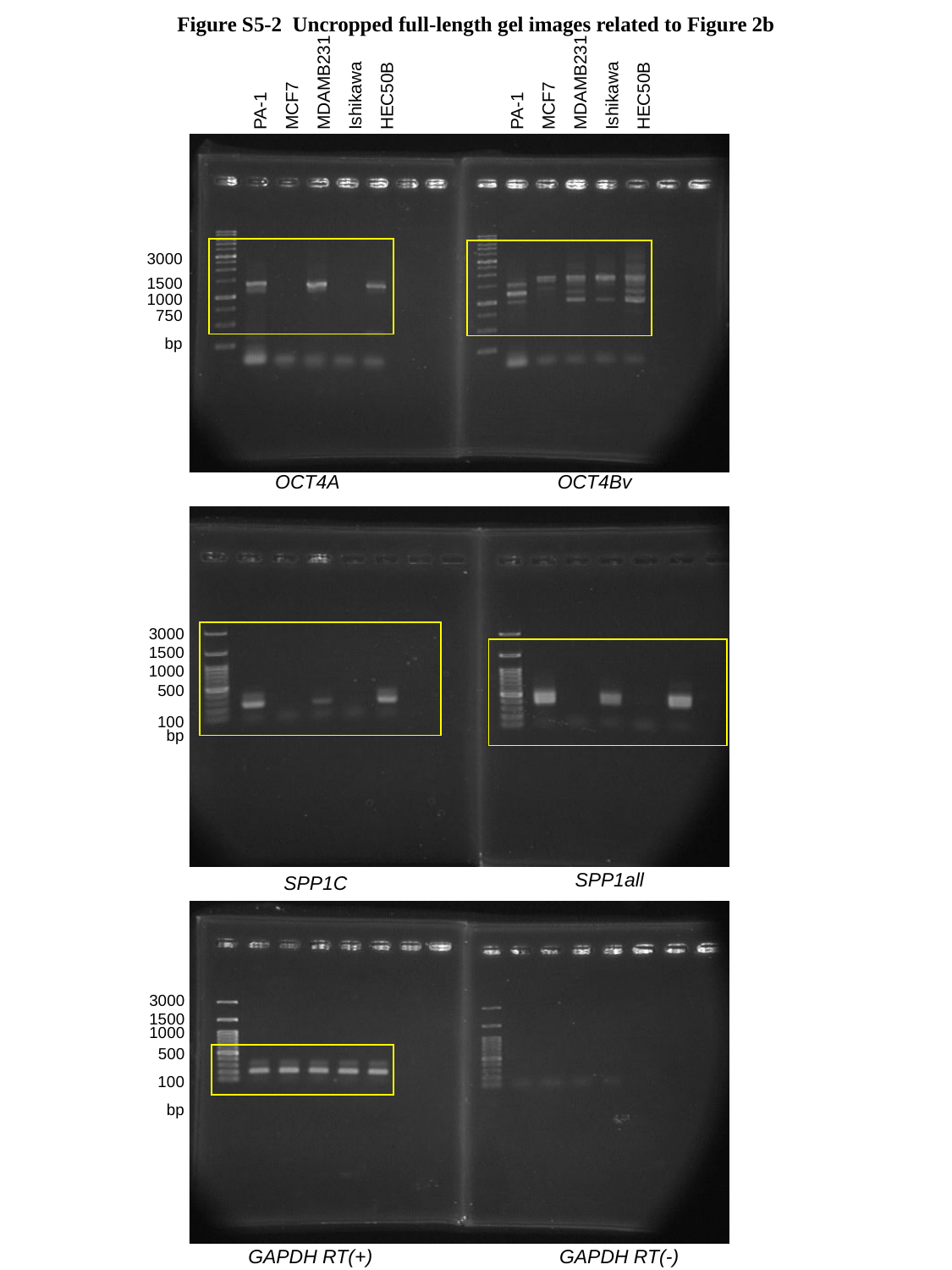

PA-1
MCF7
MDAMB231
Ishikawa
HEC50B
PA-1
MCF7
MDAMB231
Ishikawa
HEC50B
Figure S5-2 Uncropped full-length gel images related to Figure 2b
3000
1500
1000
750
bp
OCT4A
OCT4Bv
3000
1500
1000
500
100
bp
SPP1all
SPP1C
3000
1500
1000
500
100
bp
GAPDH RT(+)
GAPDH RT(-)

## Slide 11
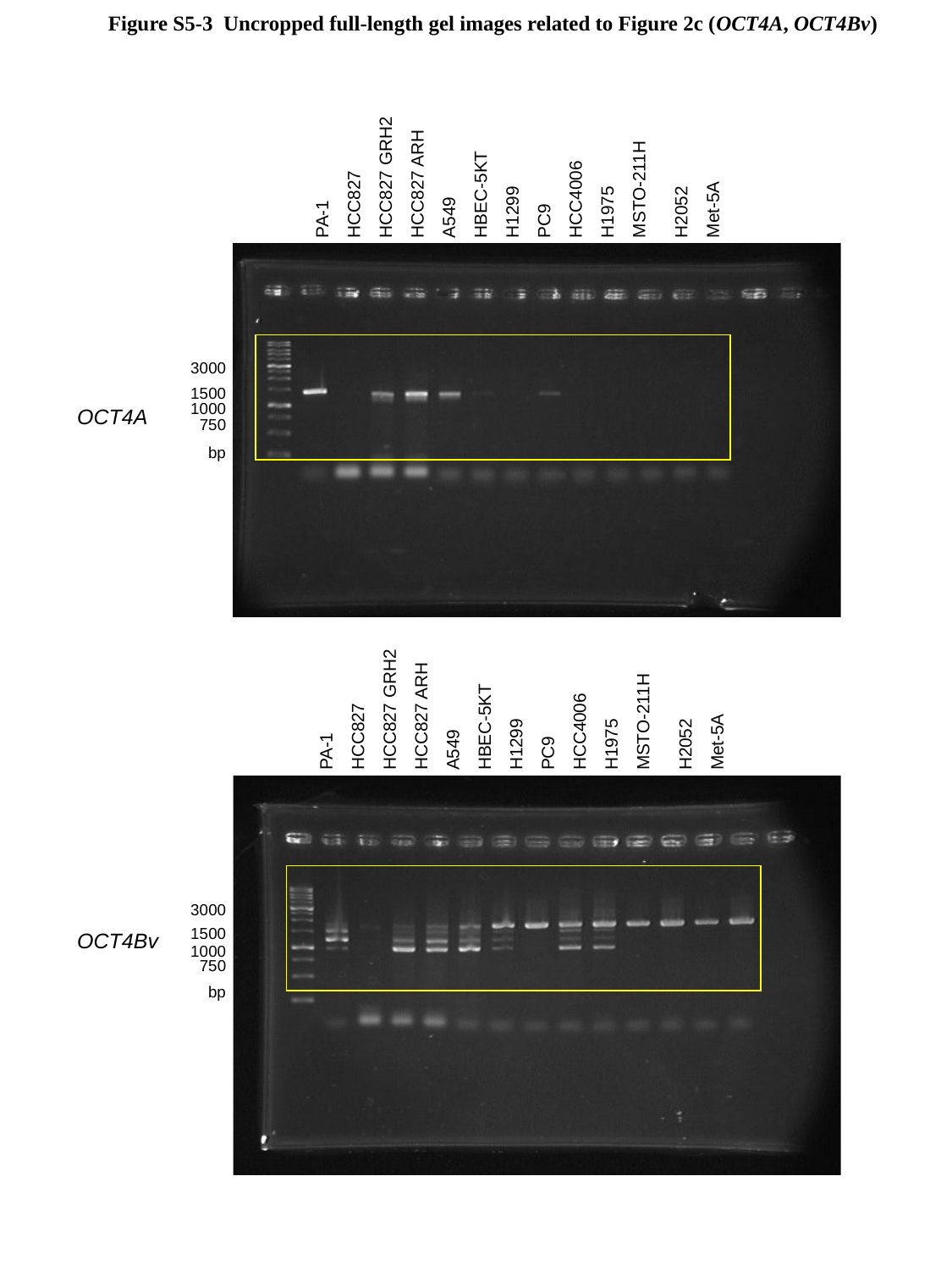

PA-1
HCC827
HCC827 GRH2
HCC827 ARH
A549
HBEC-5KT
H1299
PC9
HCC4006
H1975
MSTO-211H
H2052
Met-5A
Figure S5-3 Uncropped full-length gel images related to Figure 2c (OCT4A, OCT4Bv)
3000
1500
1000
OCT4A
750
bp
PA-1
HCC827
HCC827 GRH2
HCC827 ARH
A549
HBEC-5KT
H1299
PC9
HCC4006
H1975
MSTO-211H
H2052
Met-5A
3000
1500
OCT4Bv
1000
750
bp

## Slide 12
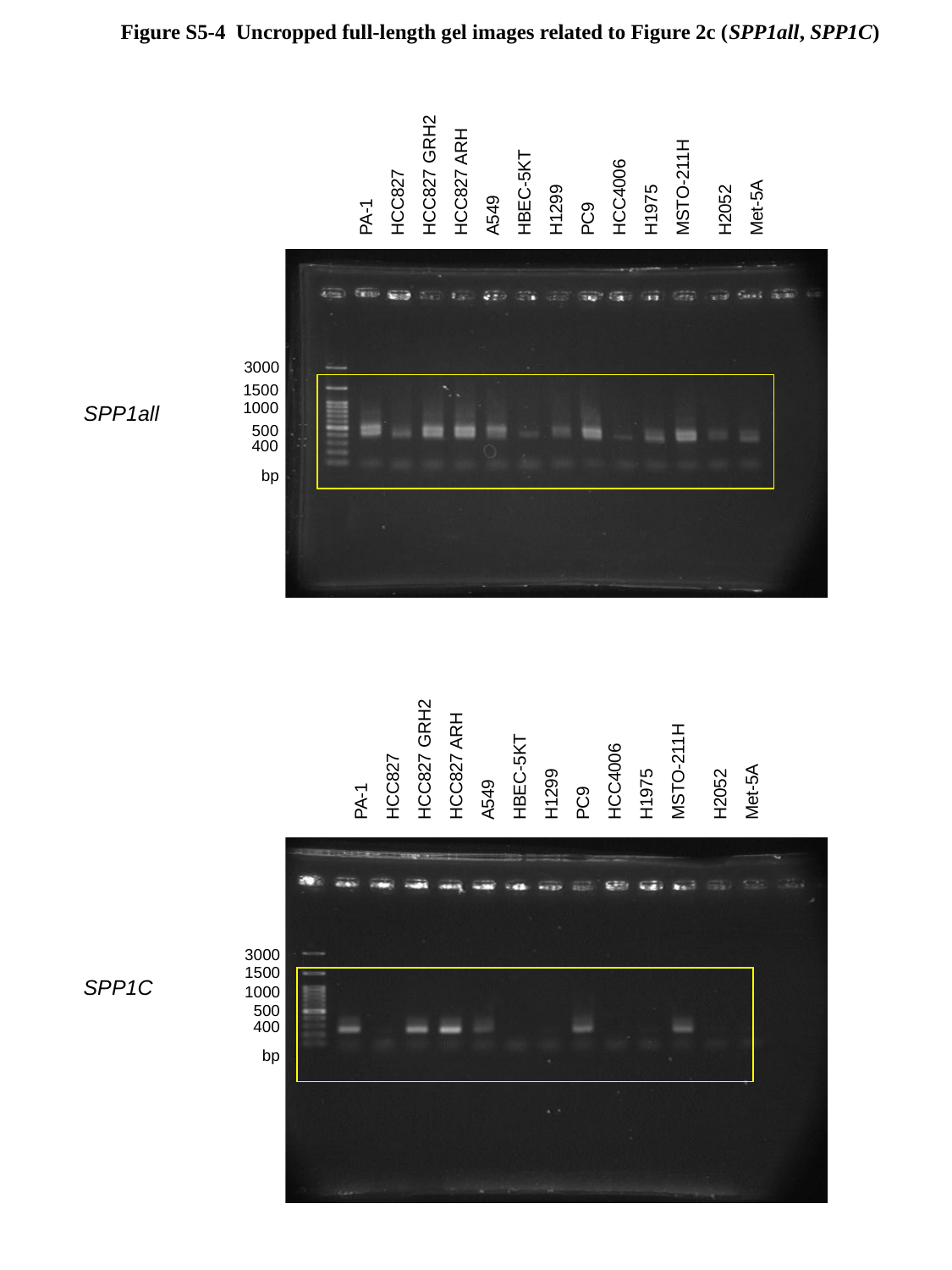

PA-1
HCC827
HCC827 GRH2
HCC827 ARH
A549
HBEC-5KT
H1299
PC9
HCC4006
H1975
MSTO-211H
H2052
Met-5A
Figure S5-4 Uncropped full-length gel images related to Figure 2c (SPP1all, SPP1C)
3000
1500
1000
SPP1all
500
400
bp
PA-1
HCC827
HCC827 GRH2
HCC827 ARH
A549
HBEC-5KT
H1299
PC9
HCC4006
H1975
MSTO-211H
H2052
Met-5A
3000
1500
SPP1C
1000
500
400
bp

## Slide 13
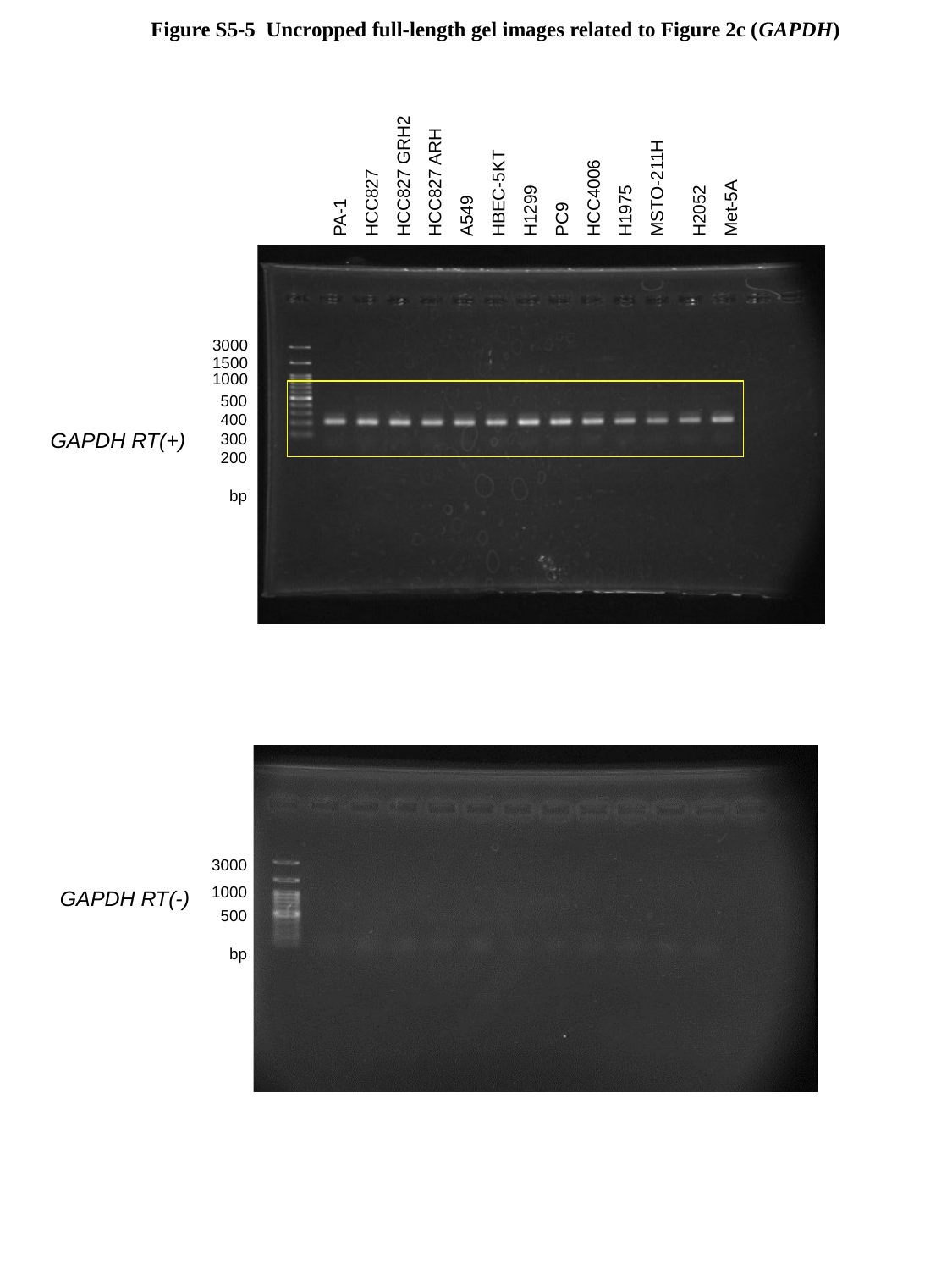

PA-1
HCC827
HCC827 GRH2
HCC827 ARH
A549
HBEC-5KT
H1299
PC9
HCC4006
H1975
MSTO-211H
H2052
Met-5A
Figure S5-5 Uncropped full-length gel images related to Figure 2c (GAPDH)
3000
1500
1000
500
400
300
200
 bp
GAPDH RT(+)
3000
1000
GAPDH RT(-)
500
 bp

## Slide 14
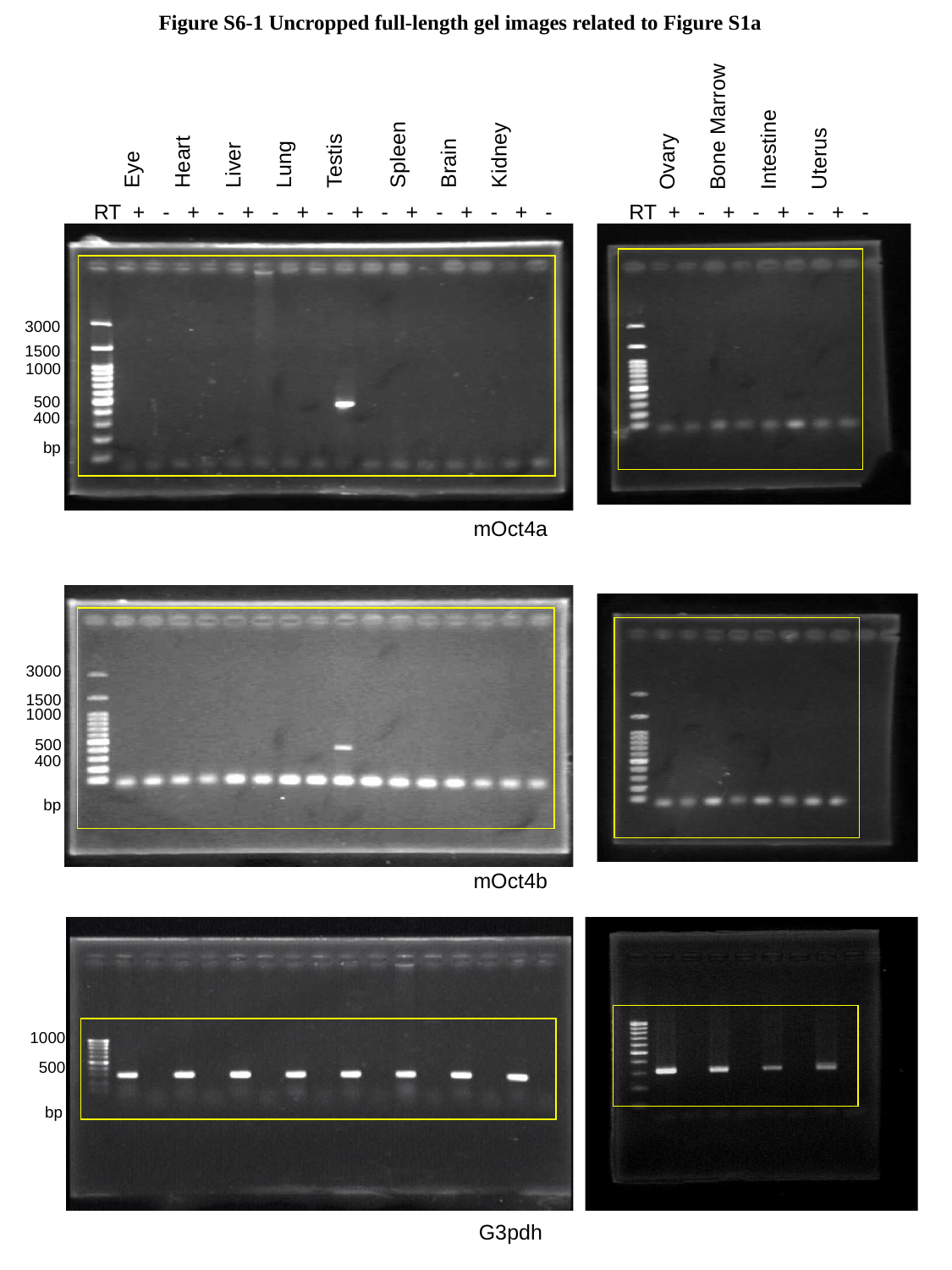

Figure S6-1 Uncropped full-length gel images related to Figure S1a
Ovary
Bone Marrow
Intestine
Uterus
Eye
Heart
Liver
Lung
Testis
Spleen
Brain
Kidney
RT + - + - + - + - + - + - + - + - RT + - + - + - + -
3000
1500
1000
500
400
bp
mOct4a
3000
1500
1000
500
400
bp
mOct4b
1000
500
bp
G3pdh

## Slide 15
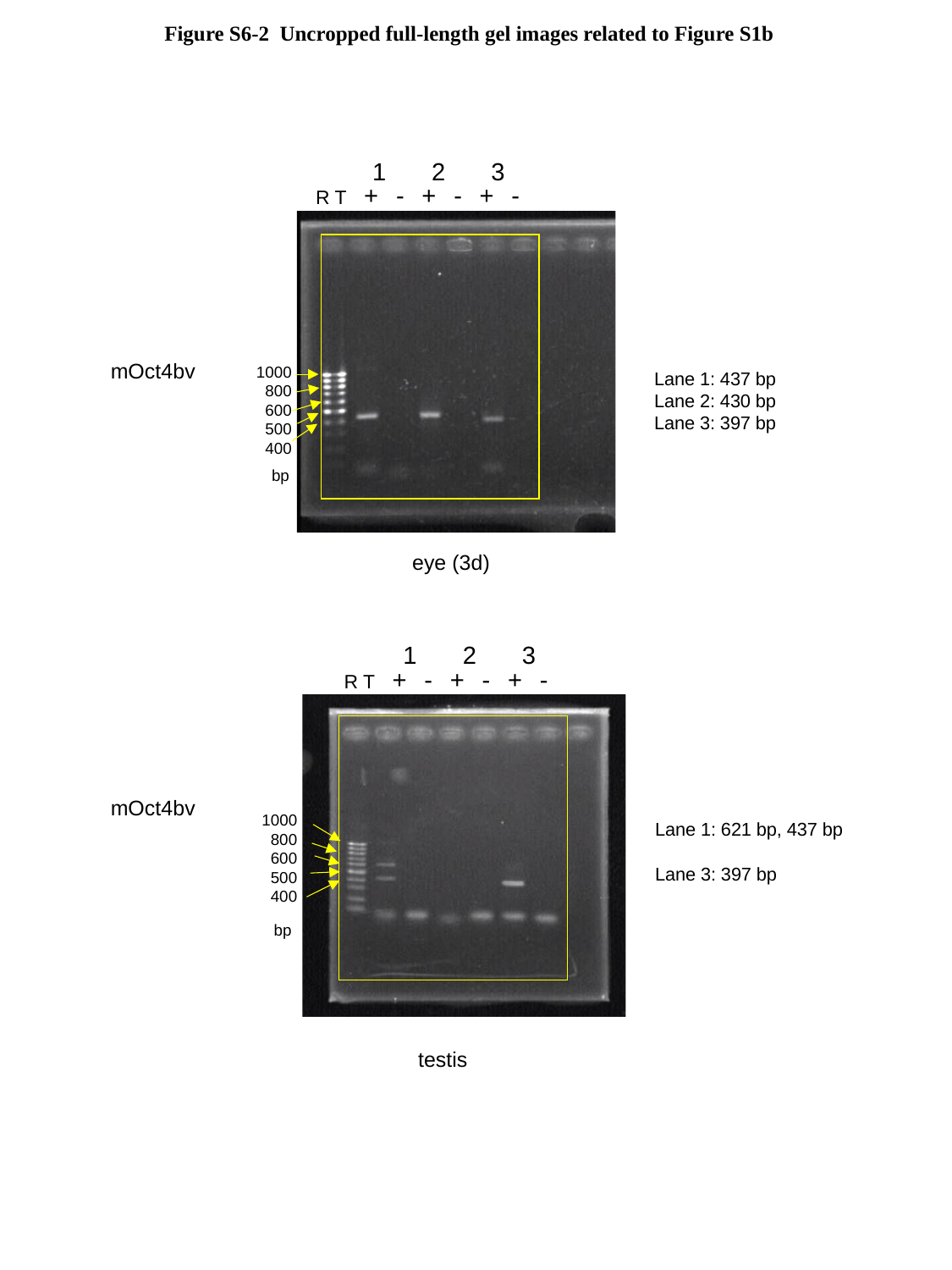

Figure S6-2 Uncropped full-length gel images related to Figure S1b
1 2 3
RT + - + - + -
mOct4bv
1000
 800
 600
 500
 400
Lane 1: 437 bp
Lane 2: 430 bp
Lane 3: 397 bp
bp
eye (3d)
1 2 3
RT + - + - + -
mOct4bv
1000
 800
 600
 500
 400
Lane 1: 621 bp, 437 bp
Lane 3: 397 bp
bp
 testis

## Slide 16
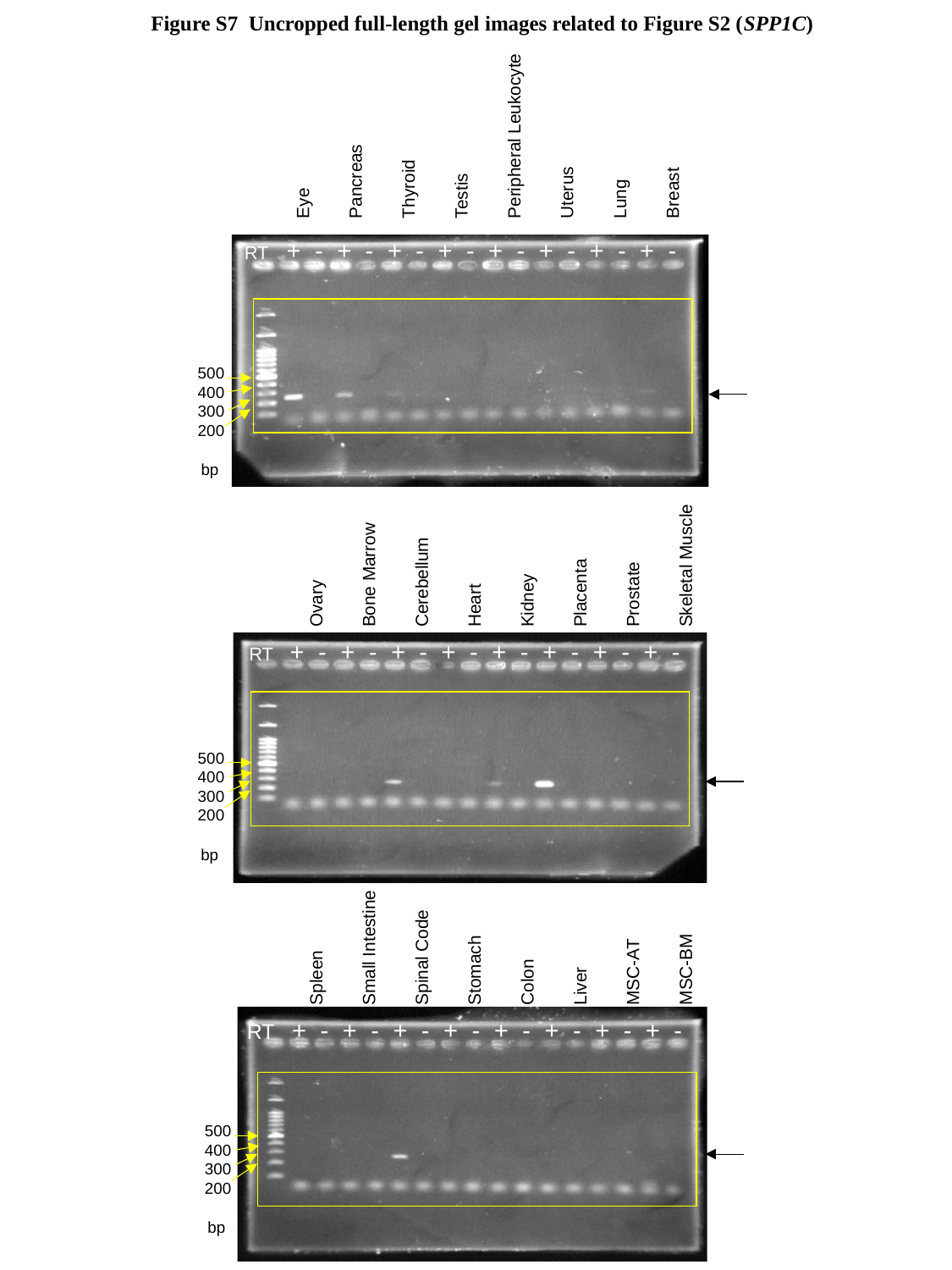

Figure S7 Uncropped full-length gel images related to Figure S2 (SPP1C)
Eye
Pancreas
Thyroid
Testis
Peripheral Leukocyte
Uterus
Lung
Breast
RT + - + - + - + - + - + - + - + -
Ovary
Bone Marrow
Cerebellum
Heart
Kidney
Placenta
Prostate
Skeletal Muscle
500
400
300
200
bp
RT + - + - + - + - + - + - + - + -
Spleen
Small Intestine
Spinal Code
Stomach
Colon
Liver
MSC-AT
MSC-BM
500
400
300
200
bp
RT + - + - + - + - + - + - + - + -
500
400
300
200
bp

## Slide 17
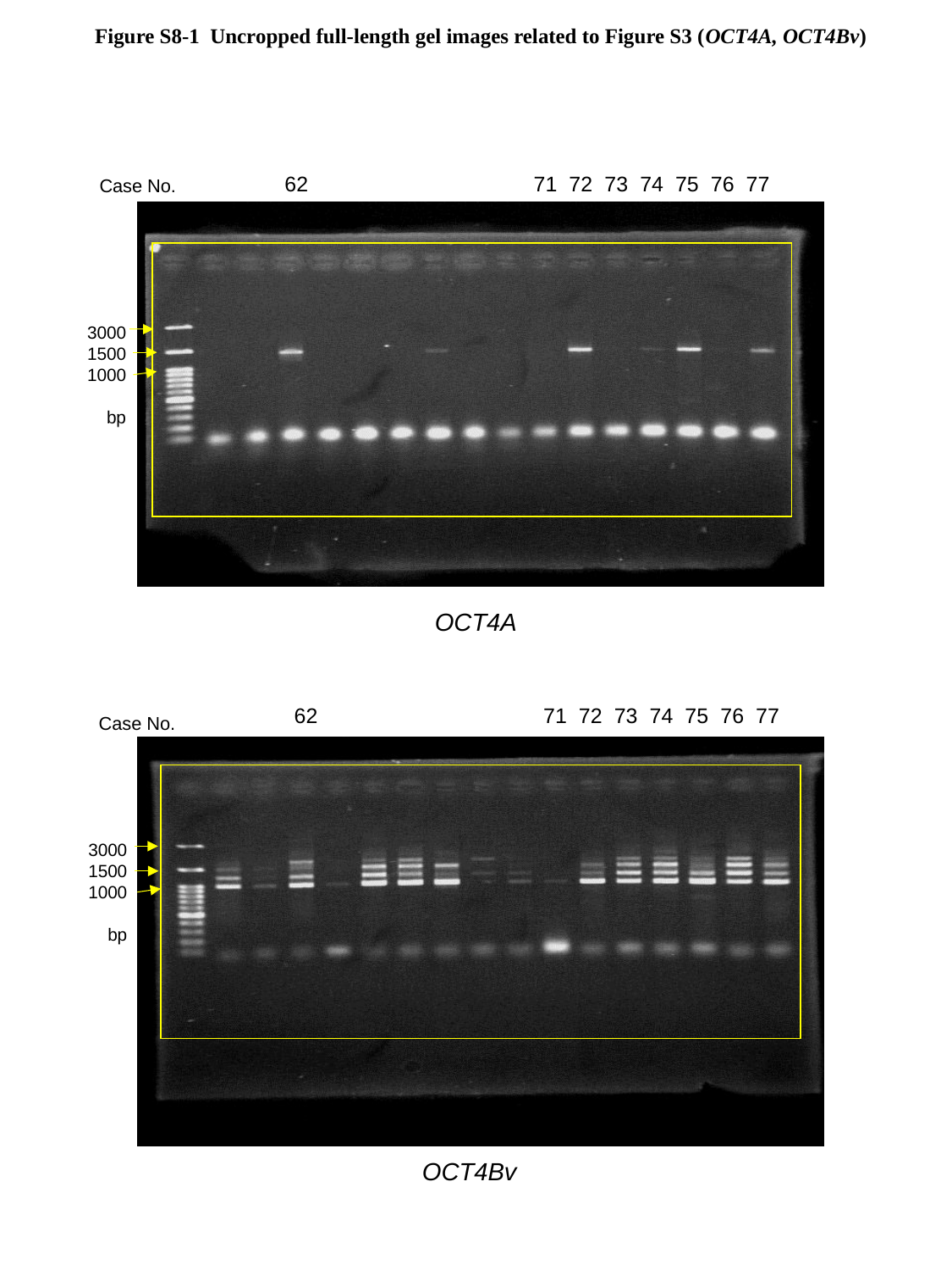

Figure S8-1 Uncropped full-length gel images related to Figure S3 (OCT4A, OCT4Bv)
62
71 72 73 74 75 76 77
Case No.
3000
1500
1000
 bp
OCT4A
62
71 72 73 74 75 76 77
Case No.
3000
1500
1000
 bp
OCT4Bv

## Slide 18
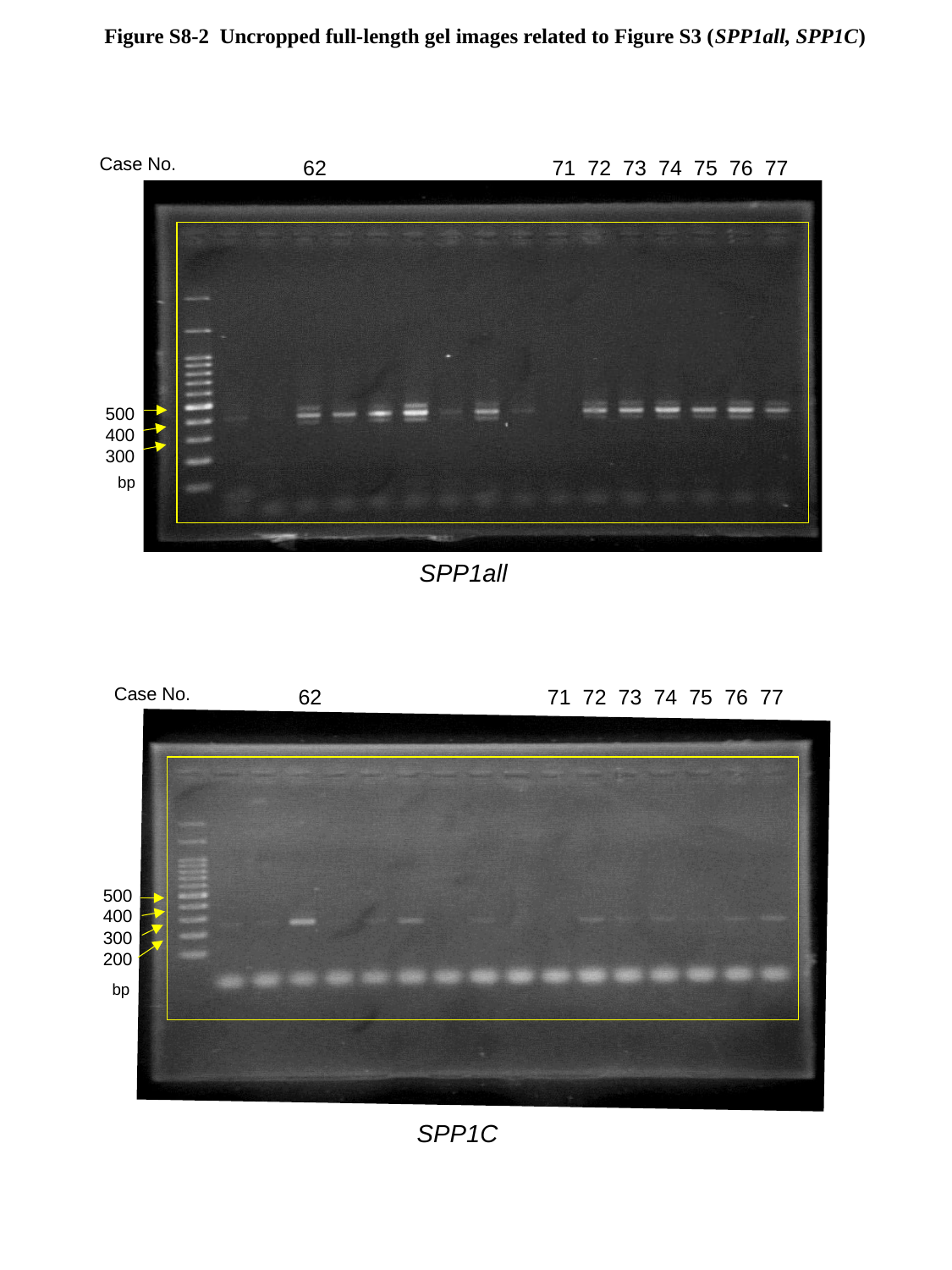

Figure S8-2 Uncropped full-length gel images related to Figure S3 (SPP1all, SPP1C)
Case No.
62
71 72 73 74 75 76 77
500
400
300
bp
SPP1all
Case No.
62
71 72 73 74 75 76 77
500
400
300
200
bp
SPP1C

## Slide 19
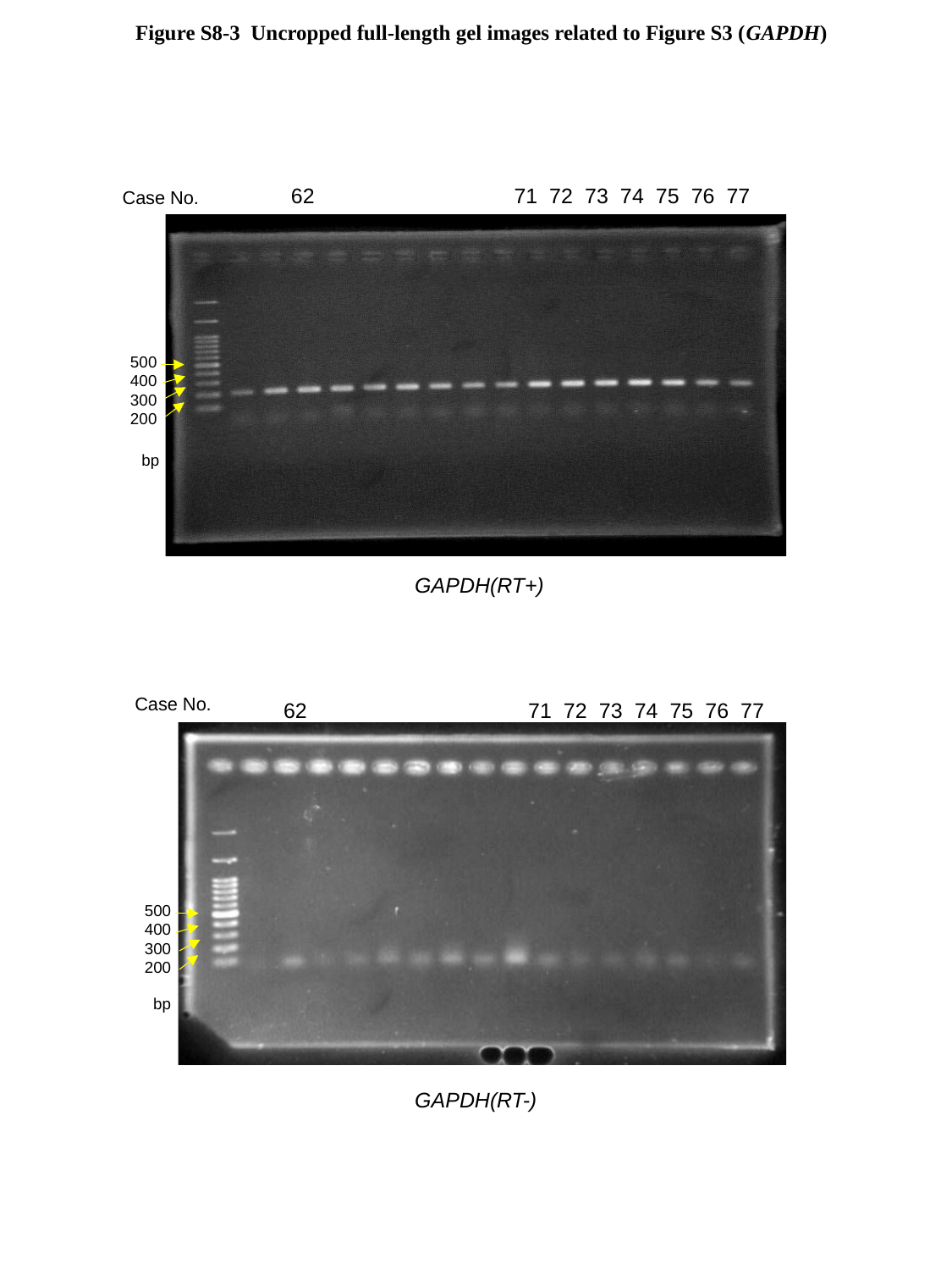

Figure S8-3 Uncropped full-length gel images related to Figure S3 (GAPDH)
62
71 72 73 74 75 76 77
Case No.
500
400
300
200
bp
GAPDH(RT+)
Case No.
62
71 72 73 74 75 76 77
500
400
300
200
bp
GAPDH(RT-)
